# Use of evidential reasoning and AHP to assess regional industrial safety

**DOI:** 10.1371/journal.pone.0197125

**Published:** 2018-05-24

**Authors:** Zhichao Chen, Tao Chen, Zhuohua Qu, Zaili Yang, Xuewei Ji, Yi Zhou, Hui Zhang

**Affiliations:** 1 Institute of Public Safety Research, Tsinghua University, Beijing, China; 2 Liverpool Business School, Liverpool John Moores University, Liverpool, United Kingdom; 3 Liverpool Logistics, Offshore and Marine Research Institute, Liverpool John Moores University, Liverpool, United Kingdom; 4 Beijing Academy of Safety Science and Technology, Beijing, China; Southwest University, CHINA

## Abstract

China’s fast economic growth contributes to the rapid development of its urbanization process, and also renders a series of industrial accidents, which often cause loss of life, damage to property and environment, thus requiring the associated risk analysis and safety control measures to be implemented in advance. However, incompleteness of historical failure data before the occurrence of accidents makes it difficult to use traditional risk analysis approaches such as probabilistic risk analysis in many cases. This paper aims to develop a new methodology capable of assessing regional industrial safety (RIS) in an uncertain environment. A hierarchical structure for modelling the risks influencing RIS is first constructed. The hybrid of evidential reasoning (ER) and Analytical Hierarchy Process (AHP) is then used to assess the risks in a complementary way, in which AHP is hired to evaluate the weight of each risk factor and ER is employed to synthesise the safety evaluations of the investigated region(s) against the risk factors from the bottom to the top level in the hierarchy. The successful application of the hybrid approach in a real case analysis of RIS in several major districts of Beijing (capital of China) demonstrates its feasibility as well as provides risk analysts and safety engineers with useful insights on effective solutions to comprehensive risk assessment of RIS in metropolitan cities. The contribution of this paper is made by the findings on the comparison of risk levels of RIS at different regions against various risk factors so that best practices from the good performer(s) can be used to improve the safety of the others.

## 1. Introduction

**Fig 1:**
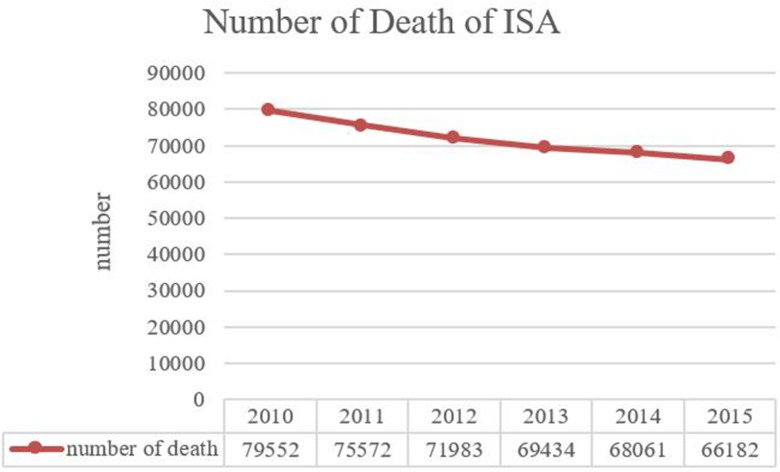
Number of death due to industrial accidents in China. Data from: National Economy and Society Developed Statistical Bulletin 2010–2015 from National Bureau of Statistics of the People’s Republic of China.

Given the rapid economic and social development, especially the fast growing industrialization and automation in a country/region, the occurrence likelihood of industrial accidents declines in general. For instance, compared with that in 2009, the number of industrial accidents in Beijing decreased by 8.2% in 2015 (Beijing Work Safety Statistical Yearbook, 2015). Fig 1 shows the number of death due to industrial accidents in China from 2010 to 2015. Although the number of death is decreasing year by year, the absolute quantities are still very large, revealing that the situation of industrial safety is severe as ever, wanting effective solutions to be found. Many researchers have made large effort to improve the industrial safety. Chryssolouris (1999) explored a virtual reality based approach for the verification of human related factors in assembly and maintenance processes [1]. Michalos (2015) made research on design consideration for safe human-robot collaborative workplaces [2]. However, ensuring industrial safety in a fast developing economy is challenging, given that major and extraordinarily serious accidents (MESA) often present low likelihoods but significant consequences. For instance, the explosion accident in Tianjin Port in 2015 caused not only a huge loss of properties and lives, but also a significant impact on industrial safety policy making, concerning the use of advanced risk analysis approaches to enhance accident prevention in the situations where hazardous events have not arisen and historical failure data has not formed any base in critical mass yet.

Because of the complicated risk factors influencing regional industrial safety (RIS), it is extremely difficult, if possible, to get all the relevant data, such as the severity of accident consequences and the occurrence probabilities of the accidents. As a result, there are few studies on regional risk assessment in the literature and fewer on use of advanced risk modelling to deal with the uncertainty in risk data. When conducting risk analysis of RIS, it is often the case that many qualitative and quantitative variables which are of high uncertainty and incompleteness in data, influence the risk level of RIS simultaneously. It is therefore necessary to develop a new method capable of tackling such challenges.

This paper aims at developing a new methodology capable of assessing RIS. Following the relevant literature review and background analysis in Section 2, a hierarchical structure for modelling the risk factors influencing RIS is first constructed in Section 3. The hybrid of evidential reasoning (ER) and Analytical Hierarchy Process (AHP) is then used to assess RIS in a complementary way in which AHP is hired to evaluate the weight of each risk factor and ER is employed to synthesise the evaluations of the risk factors from the bottom to the top level in the hierarchy. In Section 4, the hybrid approach is applied in a real case analysis of RIS across the major districts in Beijing (capital of China) to demonstrate its feasibility. Section 5 concludes the work and provides useful insights on effective solutions to comprehensive assessment of RIS in metropolitan cities.

## 2. Literature review

### 2.1 Evidential reasoning approach

An ER approach was developed in the 1990s to handle uncertainty and randomness, and is amongst the latest Multiple Criteria Decision Analysis (MCDA) techniques. It is based on the Dempster-Shafer (D-S) theory of evidence. The D-S theory that was first proposed by Dempster (1967) and developed by Shafer (1976), is regarded as a generalization of the Bayesian theory of probability. With the ability of coping with the uncertainty or imprecision embedded in evidence, the D-S theory has been widely applied in recent years [3].

ER is based on an extended decision matrix in which each attribute of an alternative is described by a distributed assessment using a belief structure. Bi et al. (2008) [4] explained that the D-S theory is an appropriate and suitable approach to dealing with uncertainty and imprecision. It provides a coherent framework to cope with the lack of evidence and discards the insufficient reasoning principle. ER enables to translate the relationship between the objects and the degree of goodness or badness of their sub-criteria, which are measured by both “the degree to which the sub-criteria are important to the objects and the degree to which the sub-criteria belong to the good (or bad) category” [5]. Furthermore, it allows decision-makers’ preference to be aggregated in a structured and rigorous way without accepting the linearity assumption [6].

Due to such advantages, ER has been widely applied to analyse the risks in various sectors when uncertainty in failure data is high. The statistics, when using the key words “evidential reasoning” and “risk” to search on web of science, shows that in 2010–2017 there are 78 journal papers (e.g. [7–19]), tackling risks in the energy, environmental, transport, offshore and logistics industries. A further in-depth analysis of these papers reveals that many of them focused on the theoretical modelling work, while the others dealing with ER’s applications in risk tend to analyse small scale cases. No studies have been found on the use of ER in RIS and to solve large scale real problems, revealing a research gap to be fulfilled, particularly from a practical perspective.

### 2.2 Analytical Hierarchy Process

AHP, developed by Saaty (1980), is proved to be a powerful tool for handling both qualitative and quantitative multi-criteria factors in solving decision-making problems. With this method, a complicated problem can be converted to an ordered hierarchical structure. The AHP method has been widely applied to multi-criteria decision making (MCDM) situations, including web site selection [20], tools’ evaluation [21], weapon selection [22], and drugs selection [23]. Its applications have also been well documented in Vaidya and Kumar, (2006) [24], Subramanain and Ramanathan, (2012) [25] in operational management, and Schmidt et al., (2015) [26] in healthcare.

The first step of AHP is to establish a hierarchical structure of presenting the problem. Then, in each hierarchical level, a nominal scale is used to construct a pairwise comparison judgement matrix. The third step is to calculate the eigenvector of the matrix. Before the eigenvector is transformed into the weights of elements, the consistency of the matrix should be checked through a consistency ratio (*CR*). If the result of *CR* is less than 0.1, the consistency of the pairwise comparison matrix M is acceptable. Consequently, the eigenvector of the pairwise comparison judgement matrix can be normalized as the final weights of decision elements. Otherwise, the consistency is not ensured and the elements in the matrix should be revised.

### 2.3 The selection of ER and AHP

AHP is a systematic technique to evaluate the relative importance between two or more attributes by means of pairwise comparisons [27]. It is able to take all of the factors into account within a hierarchic style which enables to arrange these factors systematically and to elucidate their contributions to the risks with priority weights [28]. Especially, AHP is a powerful tool for handling both qualitative and quantitative multi-criteria factors in decision-making problems [29].

The ER approach models both quantitative and qualitative attributes with uncertainty using a distributed modelling framework, in which each attribute is determined by a set of collectively exhaustive assessment grades, called a belief structure. The evidence combination rule of the D-S theory makes it possible to gather the influence of each attribute in the hierarchy. The ER approach has been widely used in effectively synthesizing pieces of evaluation from various criteria in both quantitative and qualitative forms [30].

In the risk assessment of RIS, there are many quantitative and qualitative risk factors involving high uncertainties in data. Hence, the methodology must have the capability of handling both uncertainty and quantitative and qualitative data. AHP is one of the most popular methods of assigning attribute weights with the ability to handle both qualitative and quantitative multi-criteria factors, and ER has advantage to dealing with both quantitative and qualitative attributes with uncertainty. The integration of AHP and ER approaches has been seen in many MCDM studies such as project screening, bridge condition assessment, and risk management. Zhang (2012) applied AHP combined with ER in assessing the E-commerce security. It is proved that based on the theory of AHP and ER, the model is flexible and practical to cope with qualitative, quantitative and/or uncertain factors [3]. Dehe and Bamford (2015) [31] made a comparison of the results of a MCDA model through a case of healthcare infrastructure location. It is evidenced that the solution by the combination of AHP and ER, provides a transparent and robust framework.

Although showing much attractiveness, the applications of ER and AHP in dealing with RIS, particularly to solve a large scale of real problems need yet to be investigated and validated. So the method of AHP and ER is chosen to apply in evaluating the RIS in this paper.

## 3. A new framework for risk assessment of RIS

**Fig 2:**
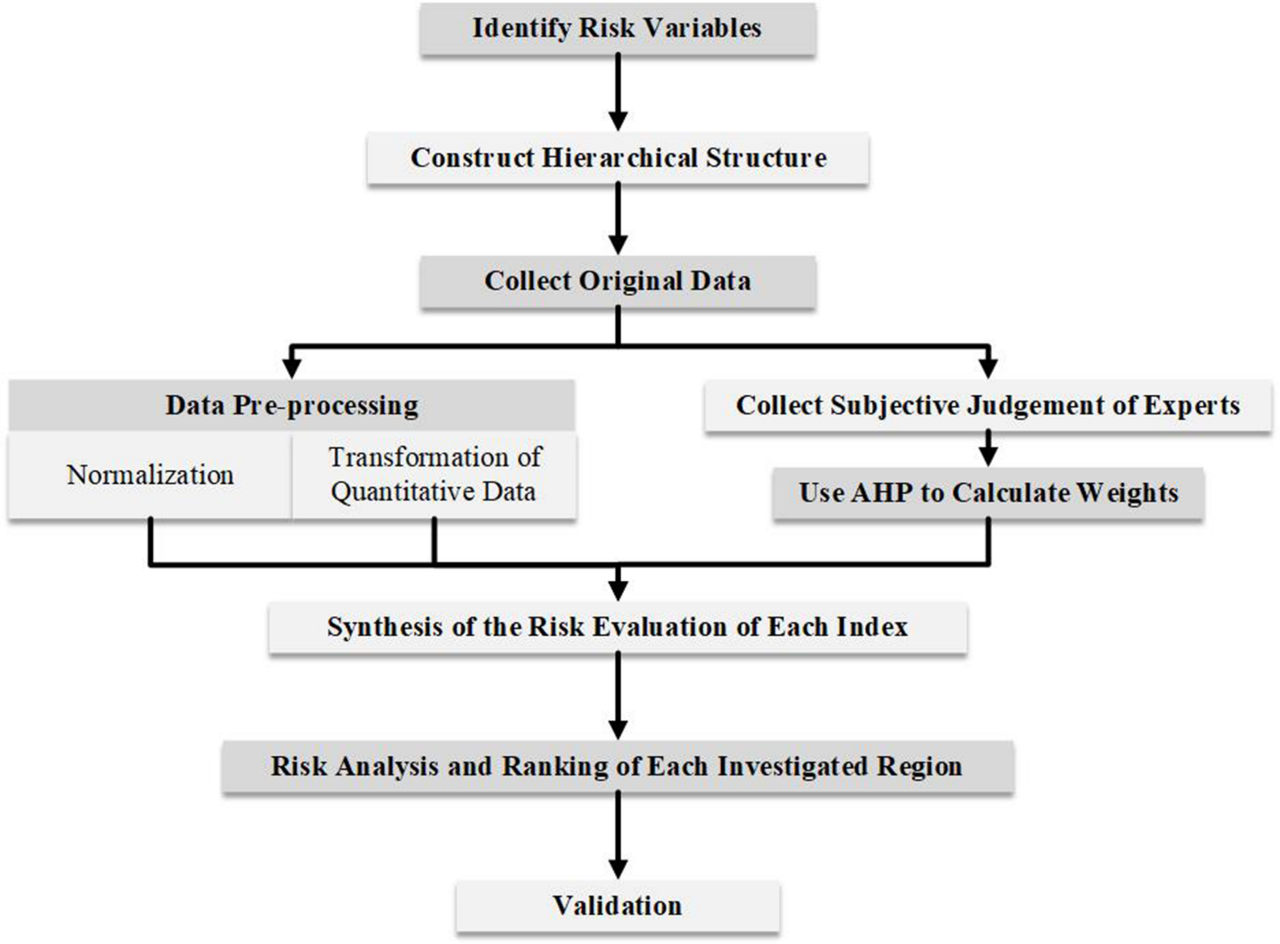
The flow chart of the framework for assessment of RIS (Source: Authors).

A flow chart is first presented in Fig 2 to visualise a new framework for risk assessment of RIS, and each of the detailed steps is described in the ensuing parts ranging from section 3.1 to section 3.5, respectively.

### 3.1 Identify risk variables and construct the hierarchical structure

The “triangular model of public safety”, proposed by Yuan et al. [32], describes public safety by three fundamental attributes, emergency, hazard-affected carriers, and emergency management. The hierarchy of evaluating RIS consists of three fundamental attributes, disaster-inducing factors, vulnerability of hazard-affected carriers, and safety control. Xie et al. (2010) [33] carried out an index system of industrial safety in Beijing, which concerned more on the historical failure data and safety supervision data, but less on the hazard-affected carriers.

Therefore, six experts possessing relevant expertise as well as representing different groups of the stakeholders were interviewed at an Expert Seminar on 12th, July, 2016 in Beijing Academy of Safety Science and Technology to go over the index system. The background information of the six persons is shown as follows.

Expert 1: A professor engaged in mining safety evaluation for more than 10 years.

Expert 2: A senior officer in China Academy of Safety Science and Technology.

Expert 3: A senior officer in Beijing Research Centre of Urban System Engineering.

Expert 4: An expert from China National Institute of Standardization.

Expert 5: A senior officer in State Administration of Work Safety.

Expert 6: An expert from Beijing Municipal Institute of Labour Protection.

The issues such as data availability, situation of industrial safety, the existing index systems were extensively discussed with the experts before having their consensus on the development of the factors influencing RIS and the hierarchical structure representing their relationship (Table 1). During this data collection process, all the participants consented to their participation in this research. The invited experts were all informed about the purpose and content of this research and any risks that might be associated with the participation prior to providing consent. We first asked for their advices about the index system by a defined questionnaire. At the Expert Seminar, the experts were consulted in verbal ways and the results were recorded in a written form (See S1 Table). This paper, together with its findings was checked by Beijing Academy of Safety Science and Technology.

**Table 1:** Comprehensive risk index system of industrial safety.

level 1	level 2	level 3	level 4
disaster-inducing factors 0.3636	accidents 0.4932	severity 0.5726	death toll of industrial safety issues 0.5403
			frequency of industrial safety issues 0.4597
		accountability 0.4274	number of people investigated and affixed liability 0.5208
			the fines of industrial safety accidents 0.4792
	hidden dangers 0.5068	number of major hazard sources 0.2664	
		number of hidden dangers discovered 0.2290	
		number of units with harm of occupational disease 0.2756	
		number of people contacted with occupational disease 0.2290	
vulnerability of hazard-affected carriers 0.3182	vulnerability 0.5333	population vulnerability 0.3371	the resident population density 0.3875
			proportion of aged population 0.2938
			proportion of children 0.3187
		infrastructural vulnerability 0.3429	number of gas station per km^2^ 1
		economical vulnerability 0.3200	the reciprocal of regional GDP per capita 0.4554
			unemployment rate 0.5446
	adaptability 0.4667	employee's assurance 0.4486	(-)number of employees joined medical assurance 0.5327
			(-)number of employees joined unemployment insurance 0.4673
		protection 0.5514	(-)investment of infrastructure 0.3494
			(-)number of medical staff per thousand people 0.3313
			(-)number of hospital beds per thousand people 0.3193
safety control 0.3182	supervision 0.5159	regulatory capacity 0.4876	(-)coverage rate of supervision 0.3558
			(-)economic punishment 0.3252
			(-)punishment rate of supervision 0.3190
		personnel allocation 0.5124	(-)crew size of safety supervision system 0.3471
			(-)number of people attending the inspection 0.2882
			(-)*capacity of the safety supervision crew 0.3647
	emergency management & publicity 0.4841	emergency capacity 0.5120	(-)number of fire brigade 0.5446
			(-)emergency resources reserves 0.4554
		safety propaganda 0.4880	(-)number of news manuscripts about industrial safety 0.4750
			(-)*the level of public safety awareness 0.5250

* symbolizes the qualitative indexes

(-) symbolizes the negative indexes

Consensus reached at the Expert Seminar on 12th, July, 2016 in Beijing Academy of Safety Science and Technology.

The numerical values in Table 1 stand for the local weight of each variable. They were calculated by using AHP.

The three risk parameters in level 1 represent the three fundamental aspects addressing the comprehensive risk of RIS. In the aspect of disaster-inducing factors, two main factors must be taken into account. One is historical accidents, and the other one is hidden dangers, which reflect the potential failures. As far as the details of accidents are concerned, severity and accountability are taken into consideration in order to reflect their relevant risk levels accordingly.

The vulnerability of hazard-affected carriers is determined by two factors, vulnerability (used to describe the easiness of an asset/a system to be destroyed) and adaptability (used to describe the difficulty of an asset/a system to be destroyed and ability that the asset/system recovers after disturbances). Vulnerability consists of population vulnerability, infrastructural vulnerability, and economical vulnerability while adaptability is associated with assurance and protection taken by the stakeholders. For instance, population vulnerability will be high if there is a large population density, high proportion of aged population and children. Adaptability will be reflected by the plan on evacuation and rescue work.

To address safety control, supervision and emergency management and publicity are taken into account. Regulatory capacity and personnel allocation are two main indexes to measure the supervision work. Similarly, emergency capacity and safety propaganda are used to represent the index of emergency management and publicity.

### 3.2 Data pre-processing

The basic input data of the indexes in level 4 are collected through a field investigation from each district in Beijing and by mining secondary data from Beijing Work Safety Statistical Yearbook, Beijing Statistical Information Net, websites of Beijing Subway and Beijing Municipal Commission of City Management.

#### 3.2.1 Normalization

Data normalization is threefold in this study. Firstly, max-min normalization is chosen to normalize the quantitative data. The initial max-min normalization process is performed using the following equation [34]:

$$t_{c}=\frac{x_{c}-x_{min}}{x_{max}-x_{min}}$$ (1)

where *x*_*c*_ represents the initial datum of district *c*, *x*_max_ and *x*_min_ represent the maximum and minimum values of the initial data associated with the same index respectively.

Secondly, the data of all the negative indexes are processed using the following equation to ensure they have the same impact on the risk contribution to the top level index.

$$r_{c}=1-t_{c}$$ (2)

The data collected by the field investigation is shown in S1 Table.

Thirdly, linguistics terms with a belief structure are employed to evaluate the qualitative indexes (i.e. capacity of the safety supervision crew and the level of public safety awareness). The 10 experts in Beijing Academy of Safety Science and Technology are interviewed to conduct the evaluation of 16 districts in Beijing based on their valuable experience which comes from their working on the frontline in the field of industrial safety using the following formula.

$$FBS=\{({FH}_{n},\beta_{n})\}$$ (3)

where *FH*_*n*_ represents the nth assessment grade; *β*_*n*_ represents the corresponding degree of belief. For instance, the five assessment grades used to define the index of “capacity of the safety supervision crew” are “Very High, High, Average, Low, Very Low”. Consequently, the S2 Table shows all the normalized data used in this research.

#### 3.2.2 Transformation of quantitative data

The normalized data of the indexes in level 4 needs to be transformed and expressed by the same utility used to describe the qualitative data in order to synthesise them for safety evaluation of the index in the top level. Fuzzy membership functions are therefore used to realise such transformation [35].

**Fig 3:**
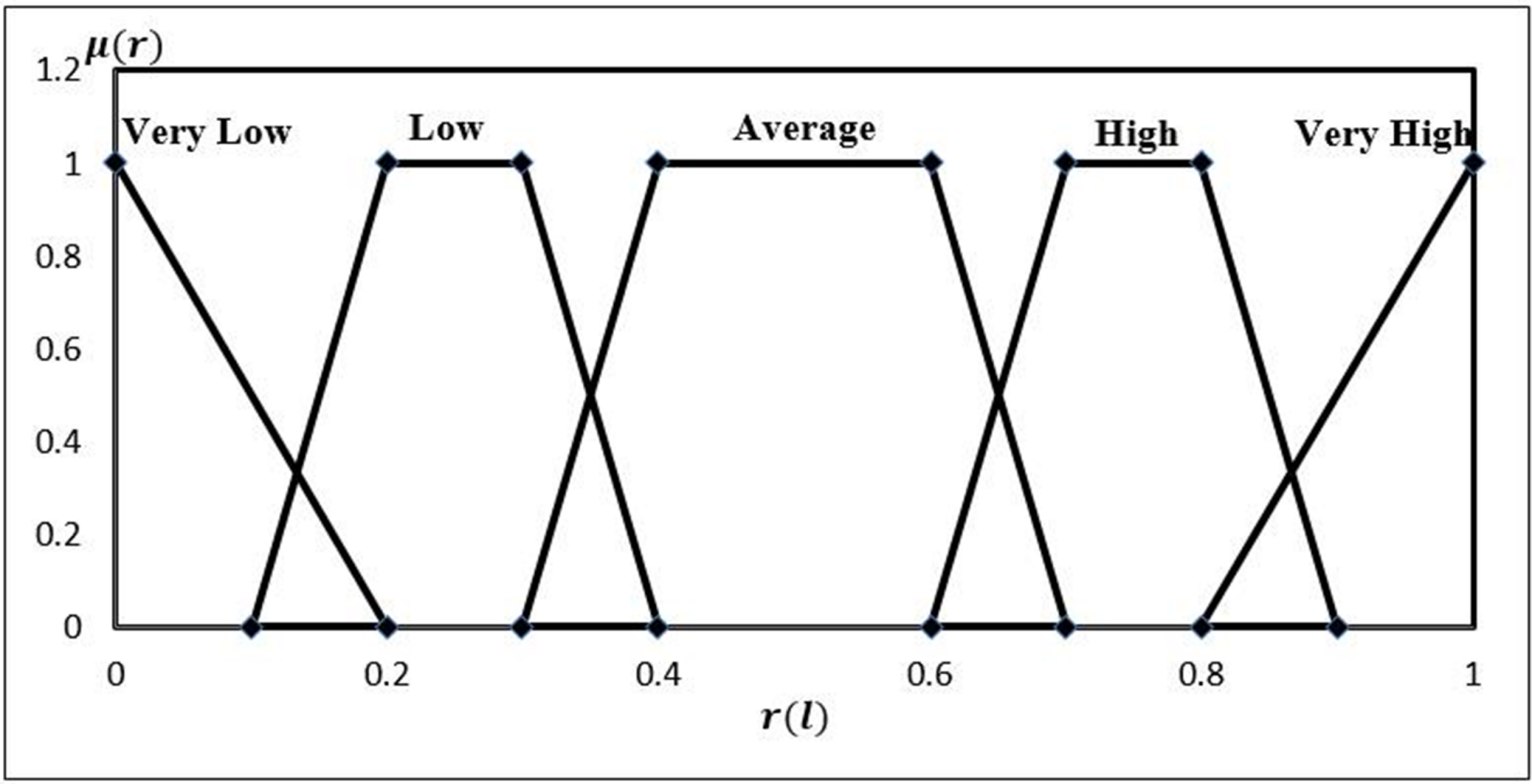
Membership function of the qualitative grades used to transform the quantitative data.

The uniformed set of qualitative grades of “Very low”, “Low”, “Average”, “High” and “Very high” and their fuzzy membership functions are defined and verified by the experts, and shown in Fig 3 [36]. It is noted here that all the quantitative data has been normalised to be associated with a crisp value in [0, 1].

**Fig 4:**
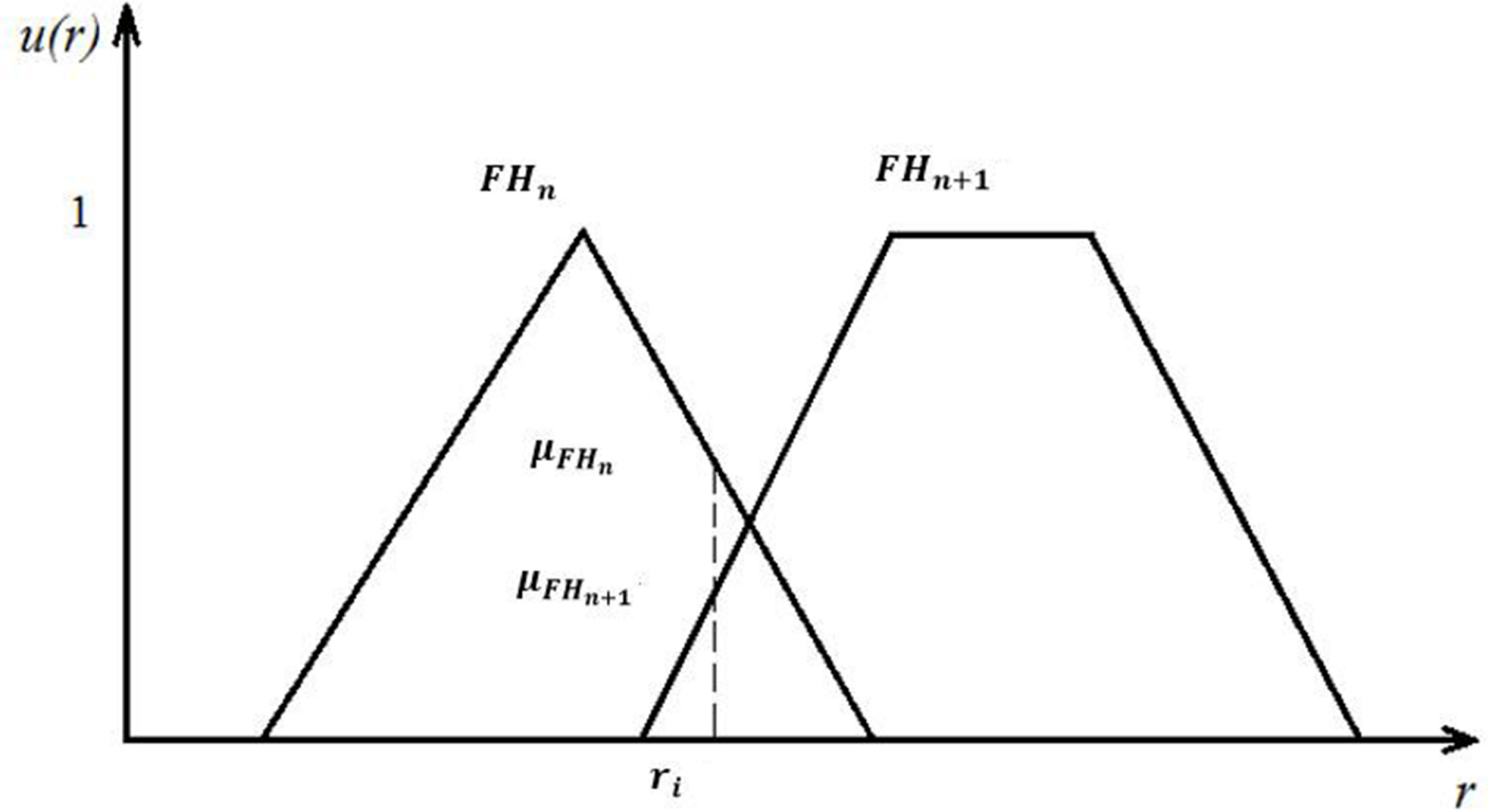
Fuzzy belief structure transforming process. * *r*_*i*_ represents the normalized value of quantitative index, and u(r) stands for the fuzzy membership, indicating the degree to which the risk value belongs to the relevant grade.

After the definition of fuzzy membership functions in Fig 3, a risk index value of a particular district can be transformed and expressed by the defined qualitative grades with a belief structure. Suppose the risk value is associated with two neighbouring grades *FH*_*n*_ and *FH*_*n*+1_, and their fuzzy memberships $\mu_{{FH}_{n}}$ and $\mu_{{FH}_{n+1}}$ indicate the degree to which the risk value belongs to the grade of *FH*_*n*_ and *FH*_*n*+1_, respectively (see Fig 4). The normalised fuzzy belief structure (FBS), *FBS* = {(*FH*_*n*_,*β*_*n*_)}, can be calculated by using Eqs (4 and 5) [37]. Consequently, all quantitative data is transformed into their qualitative counterparties, as shown in the S3 Table.

$$\beta_{n}=\frac{\mu_{{FH}_{n}}}{\mu_{{FH}_{n}}+\mu_{{FH}_{n+1}}}$$ (4)

$$\beta_{n+1}=\frac{\mu_{{FH}_{n+1}}}{\mu_{{FH}_{n}}+\mu_{{FH}_{n+1}}}$$ (5)

### 3.3 Use AHP to calculate the weights of each variable

The numerical values in Table 1 stand for the weight of each variable that is calculated by AHP. For instance, the weights of “number of major hazard sources”, “number of hidden dangers discovered”, “number of units with harm of occupational disease”, and “number of people contacted with occupational disease” are calculated as follow.

A questionnaire (S1 Questionnaire and S2 Questionnaire) was used to collect the subjective judgements of 12 experts in Beijing Academy of Safety Science and Technology. Initially 12 experts were approached because of their rich experience in the field of industrial safety management. Data from 2 experts, presenting the same evaluation of the qualitative index for all 16 districts being investigated, was found irrational and hence eliminated. All the questionnaire data of the rest 10 experts are showed in S2 Questionnaire. The grades defined in Table 2 were used by individual experts in their initial judgments in terms of the importance of the indexes. Then the average values of all the initial judgments with respect to a pair of indexes are applied into the pairwise comparison process of AHP.

**Table 2:** The standard of grading.

Importance	Grade
Unimportant	1
Slightly important	3
Fairly important	5
Obviously important	7
Absolutely important	9
Among them	2、4、6、8

The AHP matrix of the investigated four indexes is shown in Table 3.

**Table 3:** Judgement matrix.

variables	number of major hazard sources	number of hidden dangers discovered	number of units with harm of occupational disease	number of people contacted with occupational disease
number of major hazard sources (8)	1	1.1633	0.9661	1.1633
number of hidden dangers discovered (5)	0.8596	1	0.8305	1
number of units with harm of occupational disease (3)	1.0351	1.2041	1	1.2041
number of people contacted with occupational disease (3)	0.8596	1	0.8305	1

Based on the standard AHP calculations, the weights of the four indexes are obtained as 0.2664 for “number of major hazard sources”, 0.2290 for “number of hidden dangers discovered”, 0.2756 for “number of units with harm of occupational disease”, and 0.2290 for “number of people contacted with occupational disease”, respectively.

In a similar way, the weights of other indexes in Table 1 are obtained.

### 3.4 Synthesis of the risk evaluation of each index

ER can be used to synthesise the transformed risk evaluations in the S3 Table from the bottom (i.e. level 4) to the top level (i.e. level 1) in Table 1. Suppose every index *S*_*j*_ in an upper level consists of multiple (*L*) indexes in a lower level. Through the steps in Section 3.2.2, the fuzzy belief structure, *FBS*_*i*_ = {(*FH*_*n*_,*β*_*n*,*i*_)}, of every index in the lower level is acquired and expressed in S3 Table. The relevant weight of every index, *ω*_*i*_, is calculated by the method of AHP and shown in Table 1. The probability masses associated with each grade of an index in the lower level can be calculated using the following equations [38]:

$$m_{n,i}=\omega_{i}\beta_{n,i}$$ (6)

$$m_{H,i}=1-\sum_{n=1}^{N}m_{n,i}$$ (7)

$${\bar{m}}_{H,i}=1-\omega_{i}$$ (8)

$${\tilde{m}}_{H,i}=\omega_{i}(1-\sum_{n=1}^{N}\beta_{n,i})$$ (9)

where *n* = 1,2,…,*N*, representing the number of the linguistic terms, which equals to 5 in this paper; *i* = 1,2,…,*L*, representing the number of indexes in a lower level; *m*_*n*,*i*_ represents the basic belief degree to which the risk index *R*_*i*_ belongs to the grade of *FH*_*n*_; *m*_*H*,*i*_ is the unassigned probability mass caused by the lack of information, which is split into two parts, $m_{H,i}={\bar{m}}_{H,i}+{\tilde{m}}_{H,i}$; *N* represents the number of assessment grades (i.e. 5 in this study); and *L* stands for the number of indexes under the same upper index.

Next, it is to aggregate the output from *R*_*i*_ (*i* = 1,2,…,*L*) to generate the combined degree of belief of each index *S*_*j*_ at the upper level. The FBS of the index *S*_*j*_ at the upper level, ${FBS}_{S}=\{({FH}_{n},\beta_{n}^{S})\}$, can be calculated using the following equations:

$$\{H_{n}\}:m_{n,I(i+1)}=K_{I(i+1)}[m_{n,I(i)}m_{n,i+1}+m_{H,I(i)}m_{n,i+1}+m_{n,I(i)}m_{H,i+1}],\;n=1,2,\ldots ,N$$ (10)

$$m_{H,I(i)}={\bar{m}}_{H,I(i)}+{\tilde{m}}_{H,I(i)}$$ (11)

$$\left\{H\}:\;{\tilde{m}}_{H,I(i+1)}=K_{I(i+1)}[{\tilde{m}}_{H,I(i)}{\tilde{m}}_{H,i+1}+{\bar{m}}_{H,I(i)}{\tilde{m}}_{H,i+1}+{\tilde{m}}_{H,I(i)}{\bar{m}}_{H,i+1}\right]$$ (12)

$$\left\{H\}:\;{\bar{m}}_{H,I(i+1)}=K_{I(i+1)}[{\bar{m}}_{H,I(i)}{\bar{m}}_{H,i+1}\right]$$ (13)

$$K_{I(i+1)}={\left\{1-\sum_{t=1}^{N}\sum_{\begin{array}{c} j=1\\ j\neq t \end{array}}^{N}m_{t,I(i)}m_{j,i+1}\right\}}^{-1}\;i=\{1,2,\ldots ,L-1\}$$ (14)

$$\beta_{n}^{S}=\frac{m_{n,I(L)}}{1-{\bar{m}}_{H,I(L)}},\;n=1,2,\ldots ,N$$ (15)

$$\beta_{H}^{S}=\frac{{\tilde{m}}_{H,I(L)}}{1-{\bar{m}}_{H,I(L)}}$$ (16)

where *m*_*n*,*I*(*i*)_ (*n* = 1,2,…,*N*), ${\tilde{m}}_{H,I(i)}$ and ${\bar{m}}_{H,I(i)}$ denote the combined probability masses generated by aggregating the first *i* indexes.

Through Eqs (10)–(16), the belief structure of the index *S*_*j*_ is obtained. $\beta_{n}^{S}$ means the likelihood to which *H*_*n*_ is assessed. $\beta_{H}^{S}$ is the unassigned degree of belief representing the extent of incompleteness in the overall assessments. Similarly, the generated assessment for *S*_*j*_ can be represented by the following distribution:

$$S_{j}=\{(H_{n},\beta_{n}^{S}),\{n=1,2,\ldots ,N\}$$

where *S*_*j*_ is assessed to the grade *H*_*n*_ with the degree of belief of $\beta_{n}^{S}(n=1,2,\ldots ,N)$.

Such a process continues from the bottom to the top level along the hierarchy (in Table 1) until the *FBS* of the index at the top level is acquired.

### 3.5 Risk analysis and ranking of each investigated region

Through the steps in Section 3.4, the *FBS* of each index at all the four levels (in Table 1) can be calculated and expressed by the defined grades with a belief structure. To prioritise the investigated regions in terms of their risks, utility values *u*(*H*_*n*_), are assigned in a linear form (i.e. 0, 0.2, 0.4, 0.6, 0.8, 1) to the five defined grades [39], respectively. Consequently, the crisp risk score of each investigated region can be computed using Eq (17).

$$u(E)=\sum_{n=1}^{N}\beta_{n}u(H_{n})$$ (17)

where *N* denotes the number of the linguistic terms; and N equals to 5 in this paper.

### 3.6 Validation

A sensitivity analysis is conducted to test the proposed risk assessment framework of RIS. Sensitivity analysis refers to analysing how sensitive the conclusions are to minor changes in inputs [39]. If the methodology is sound, the sensitivity analysis must, at least, follow the following three axioms.

**Axiom 1:** A slight increment/ decrement in the degrees of belief associated with any linguistic variables of the lowest-level factors will certainly result in the effect of a relative increment/decrement in the result of industrial safety risk assessment of each district.

**Axiom 2:** Given the same variation of belief degree distributions of the lowest-level factors, its influence magnitude to the result of industrial safety risk assessment of each district will keep consistency with their weight distributions.

**Axiom 3:** The total influence magnitude of *x* factors (evidence) in the lowest level on the result of industrial safety risk assessment of each district will be always greater than the one from the set of *x* − *y* (*y* ∈ *x*) factors (subevidence).

To validate the methodology, a new method of sensitivity analysis [40] is applied in this case study. First, a belief degree of 0.1 belonging to the grade(s) of the highest risk contributions (e.g. “Very high” and “High”) is reassigned and moved toward the maximal decrement of risk of industrial safety at a step of 0.01 to obtain the Low Risk Inference (*LRI*), which is calculated using the following equation:

$$LRI={Risk}_{initial}-{Risk}_{after\;change}$$ (18)

Next, similarly, a belief degree of 0.1 belonging to the grade(s) of the lowest risk contributions (e.g. “Very low” and “Low”) is reassigned and moved toward the maximal increment of risk of industrial safety at a step of 0.01 to obtain the High Risk Inference (*HRI*), which is calculated using the following equation:

$$HRI={Risk}_{after\;change}-{Risk}_{initial}$$ (19)

where *Risk*_*initial*_ stands for the initial industrial safety risk based on the initial *FBS*s; *Risk*_*after change*_ stands for the industrial safety risk after the change of *FBS*s in Eqs (18) and (19).

Lastly, the average value will show the True Risk Influence (*TRI*) of each index, which can be calculated as follows:

$$TRI=\frac{LRI+HRI}{2}$$ (20)

## 4. Comprehensive risk assessment of RIS in Beijing

### 4.1 Study areas

Due to its rapid industrialization, Beijing, 39°26′N − 41°03′N, 115°25′E − 117°30′E, the capital of China, is facing lots of challenges on ensuing its industrial safety. The occurrence of any major industrial safety accident could cause huge loss in terms of both human lives and financial costs. In this real case study, the 16 districts in Beijing are investigated to assess the comprehensive risks in order to improve their RIS. Through a comparative study of different districts, the vulnerability of each district in terms of the industrial safety related work are identified to aid the governments on risk based safety decisions.

### 4.2 Application of the new methodology to the case

**Fig 5:**
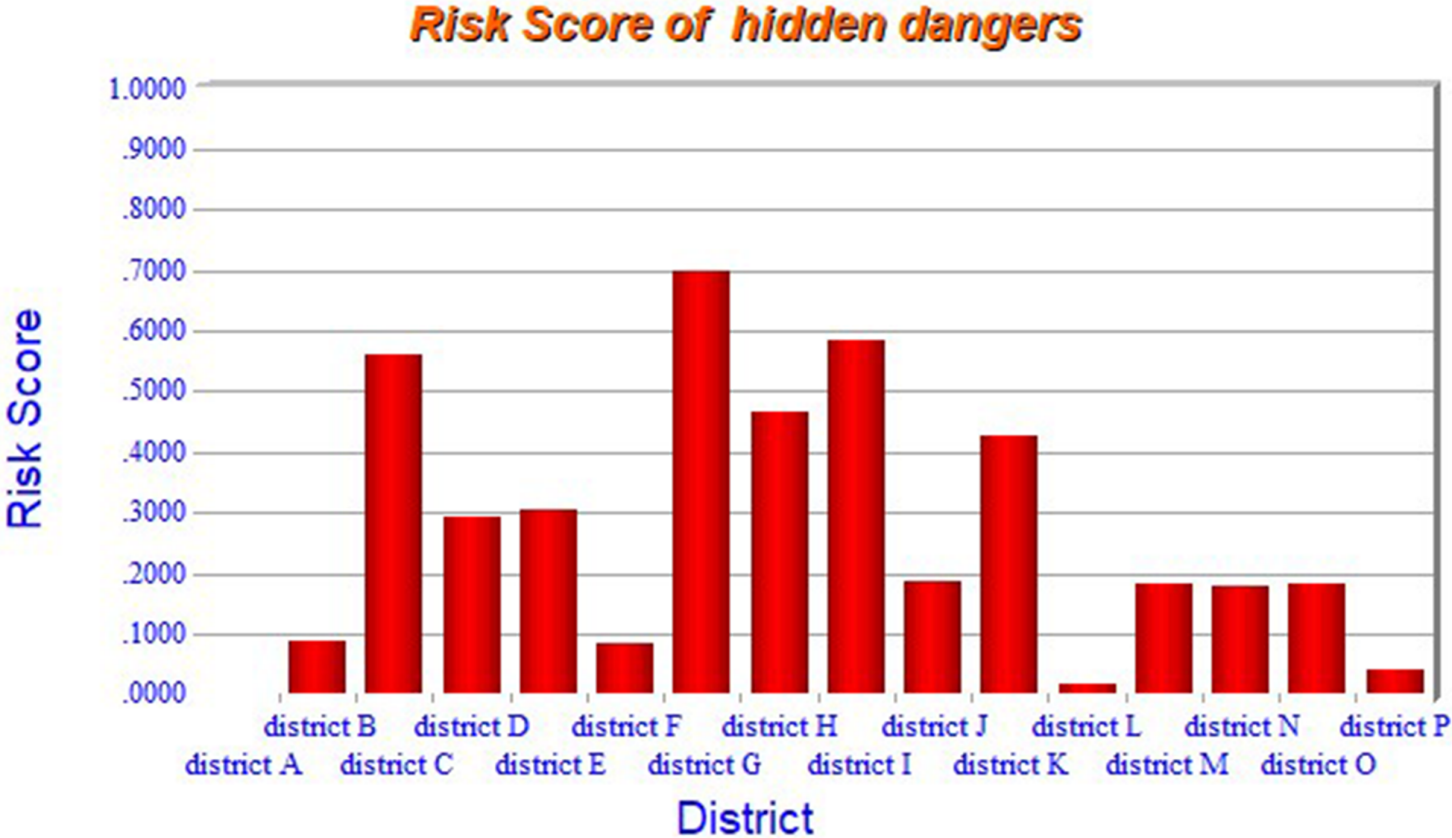
Assessment result of hidden dangers of each district.

From Table 1, it is known that hidden dangers are influenced by four indexes of “number of major hazard sources”, “number of hidden dangers discovered”, “number of units with harm of occupational disease”, and “number of people contacted with occupational disease”. Given the weights of the four indexes (in Table 1) and the risk evaluation of each district with respect to the four indexes (in S3 Table), the ER algorithm (i.e. Eqs 10–16) are used to calculated the risk score of each district in terms of hidden dangers. Using the ER associated computing software IDS [39], the risk score of each district in terms of their hidden dangers, is shown as Fig 5.

### 4.3 The result of assessment

**Fig 6:**
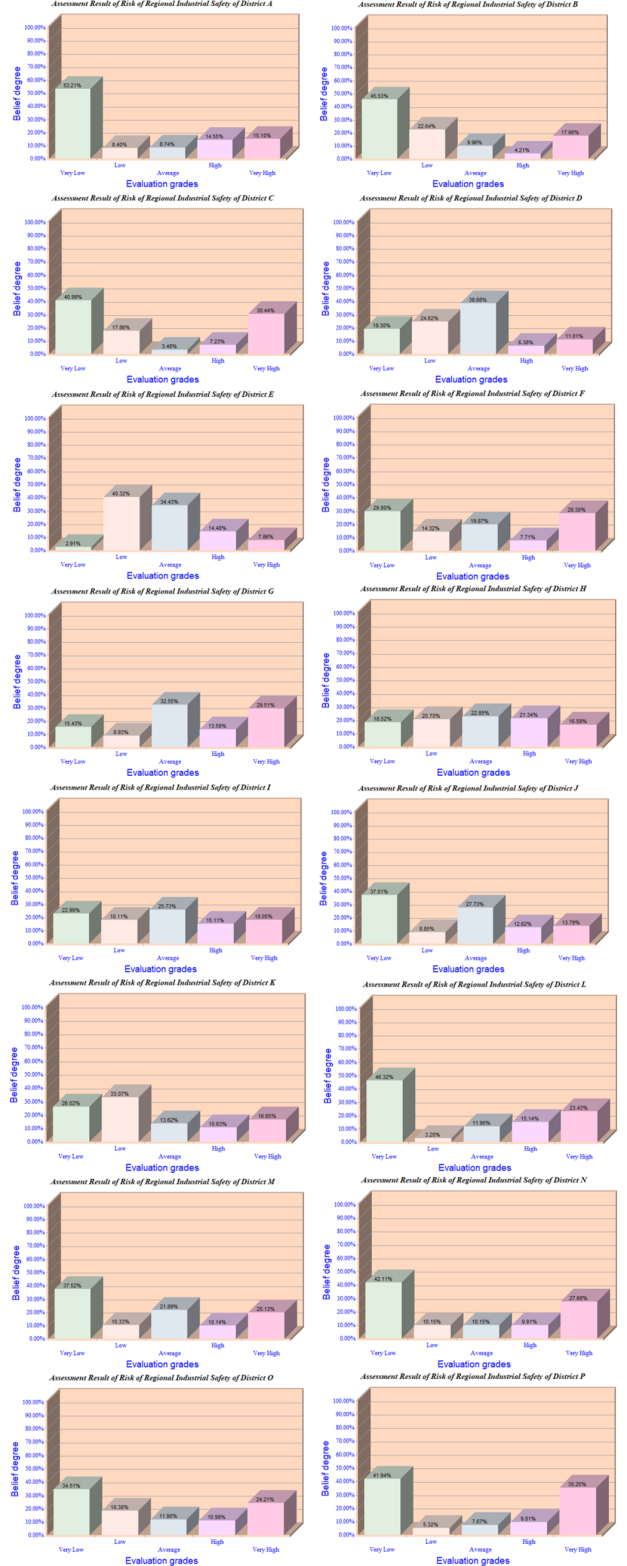
Result (FBS) of industrial safety comprehensive risk assessment of each district.

Similar to the analysis in Section 4.2, the final risk score of each investigated district is calculated by using the IDS software to produce the results graphically. It is seen in Fig 6.

Consequently, the result of district A is

{(VeryLow,53.21%),(Low,8.40%),(Average,8.74%),(High,14.55%),(VeryHigh,15.10%)}.

It means that the risk of industrial safety in district A is 53.21% “Very Low”, 8.40% “Low”, 8.74% “Average”, 14.55% “High”, and 15.10% “Very High”. Given that 61.61% belongs to “Very low” and “Low”, district A’s industrial safety situation is relatively good.

**Fig 7:**
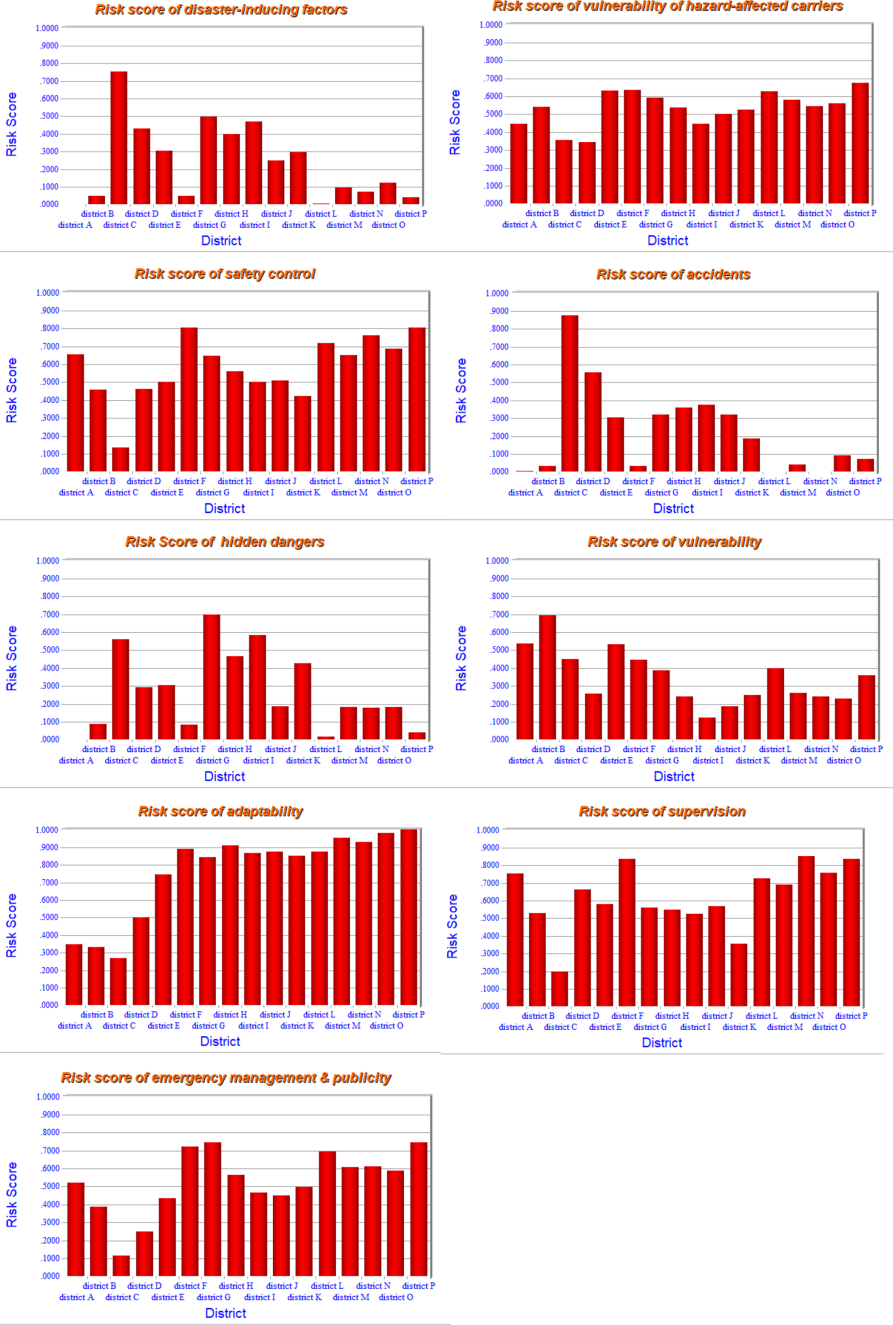
The result of each part of the hierarchy.

Next Eq 17 is used to calculate the risk score of each district with respect to different indexes. The assessment result of each district with respect to an index at any level of the hierarchy can be calculated and is showed in Fig 7.

From Fig 7, the strengths and weaknesses of each district can be clearly observed. For example, for district A, the figures above show that its vulnerability and supervision are of high risk. In other words, its vulnerability is high and its supervision related work has not been undertaken well. It is wise and necessary for the government of district A to put more effort and resources to improve it.

**Fig 8:**
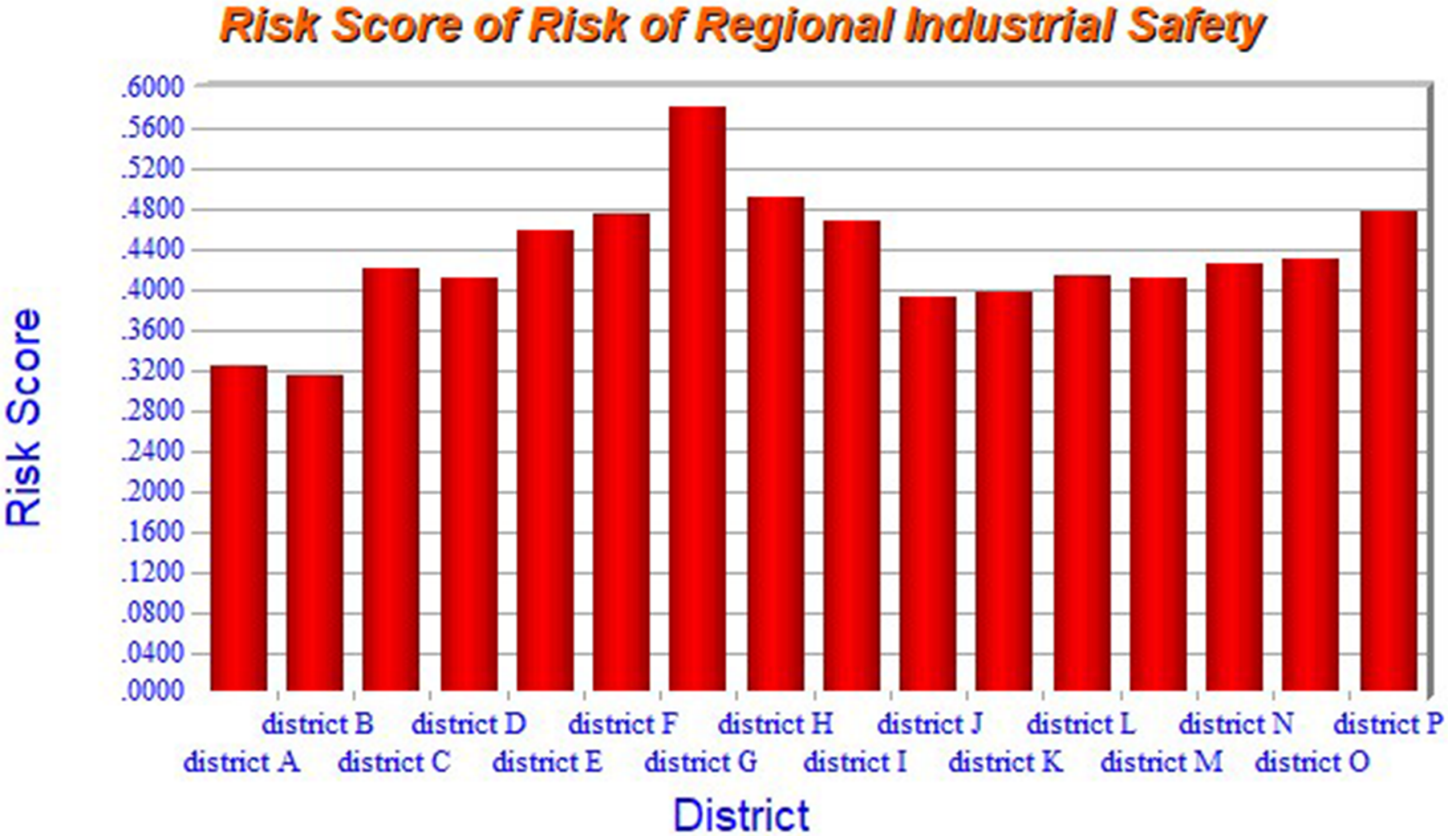
The final assessment result of risk score for each district.

Finally, the total comprehensive risk score of each district by taking into account all the indexes is obtained and shown in Fig 8.

From Fig 8, it is clear that the comprehensive risk of district G in terms of industrial safety is the highest, while the one in district B is the lowest.

The results in Fig 8 provide useful insights on which district possesses the highest level of industrial safety and which aspects of security work should be enhanced. All of these results possess an important value to both governments and the related enterprises.

### 4.4 Sensitivity analysis

To validate the methodology, a sensitivity analysis is carried out. Because of the number of the variables, it is impracticable to apply sensitivity analysis to all variables. According to the highest weight distribution, a branch of the hierarchy is chosen to be a representation, as showed in Table 4.

**Table 4:** A branch chosen to conduct the sensitivity analysis.

number of major hazard sources
number of hidden dangers discovered
number of units with harm of occupational disease
number of people contacted with occupational disease

After the input data transformation, the risk evaluations are expressed by *FBS*s such as (1, 0, 0, 0, 0), and (0, 0, 0, 0, 1), using the method which is mentioned in section 3.6, the results of the sensitivity analysis are shown as followed.

**Fig 9:**
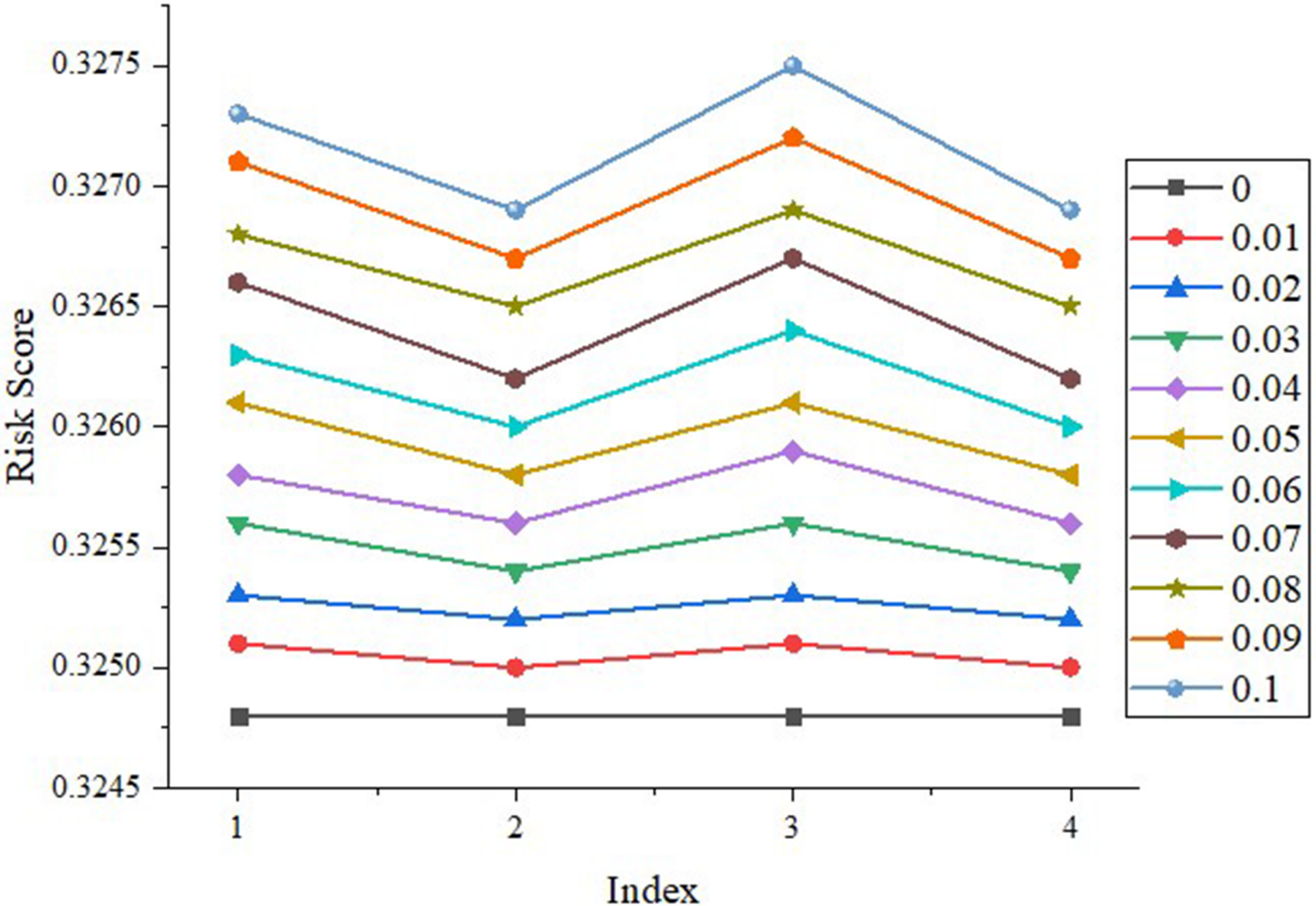
Sensitivity analysis of a branch of hierarchy of district A. *Note*: 1 stands for number of major hazard sources; 2 stands for number of hidden dangers discovered; 3 stands for number of units with harm of occupational disease; and 4 stands for number of people contacted with occupational disease.

First, because the *FBS*s of these four indexes of district A are all (1, 0, 0, 0, 0), a change of belief degree from 0 to 0.1 with a step of 0.01 is used for each variable toward the maximal increment of risk of industrial safety. Then, the risks are calculated and showed in Fig 9.

Then, districts A to D are taken as examples to calculate the true risk inference (*TRI*).

First, all the results obviously keep harmony with *Axiom* 1 in section 3.4. That is to say, the industrial safety of each district is sensitive to the variation of the lowest-level factors. Fig 9 shows the influence magnitude based on the weight distribution. A change of belief degree from 0 to 0.1 with a step of 0.01 is used for each variable toward the maximal increment of risk of industrial safety. The result reveals that it is consistent with *Axiom* 2 in section 3.4.

Then the results in Tables 5–7 show that the total influence magnitude of *x* factors in the lowest level on the result of risk assessment of each district will be always greater than the one from the set of *x* − *y* (*y* ∈ *x*) factors, which means that it keeps consistent with Axiom 3 in section 3.4. It can be easily examined by comparing the risk of districts in the chosen row in Table 7. For instance, Row 12 is chosen as the evidence, and Rows 2, 3, 4, 6, 7, 9 are identified as the sub-evidence. Comparing all the industrial safety risks of district A (i.e., the TRI of district A in Row 12 is 0.00395, which is larger than that in Rows 2, 3, 4, 6, 7, and 9), it indicates that the model is validated through the investigation of Row 12. Similarly, a comparison of all the results in Table 7 has also been examined.

**Table 5:** High Risk Inference (*HRI*).

Row	I	II	III	IV	*HRI* OF RISK OF DISTRICT			
					A	B	C	D
1	0	0	0	0	0	0	0	0
2	1	0	0	0	0.0025	0.0042	0.0073	0.0049
3	0	1	0	0	0.0021	0.0021	0	0.0043
4	0	0	1	0	0.0027	0.0045	0.0026	0.0022
5	0	0	0	1	0.0021	0.0034	0.0068	0.0016
6	1	1	0	0	0.0048	0.0065	0.0073	0.0093
7	1	0	1	0	0.0055	0.009	0.0098	0.0073
8	1	0	0	1	0.0048	0.0079	0.0138	0.0067
9	0	1	1	0	0.0049	0.0068	0.0026	0.0066
10	0	1	0	1	0.0043	0.0057	0.0068	0.006
11	0	0	1	1	0.0049	0.0082	0.0094	0.0039
12	1	1	1	0	0.0079	0.0115	0.0098	0.0118
13	1	1	0	1	0.0072	0.0103	0.0138	0.0112
14	1	0	1	1	0.0079	0.013	0.0163	0.0092
15	0	1	1	1	0.0074	0.0107	0.0094	0.0085
16	1	1	1	1	0.0106	0.0156	0.0163	0.0139

Note: "1" means that a 0.1 degree of belief is reassigned and move toward the maximal increment of risk of industrial safety of each district.

**Table 6:** Low Risk Inference (*LRI*).

Row	I	II	III	IV	*LRI* OF RISK OF DISTRICT			
					A	B	C	D
1	0	0	0	0	0	0	0	0
2	1	0	0	0	0	0	0	0
3	0	1	0	0	0	0.0019	0.0063	0.0012
4	0	0	1	0	0	0.0017	0.0081	0.0037
5	0	0	0	1	0	0	0.0021	0.0032
6	1	1	0	0	0	0.0019	0.0063	0.0012
7	1	0	1	0	0	0.0017	0.0081	0.0037
8	1	0	0	1	0	0	0.0021	0.0032
9	0	1	1	0	0	0.0035	0.0145	0.0049
10	0	1	0	1	0	0.0019	0.0085	0.0044
11	0	0	1	1	0	0.0017	0.0103	0.0069
12	1	1	1	0	0	0.0035	0.0145	0.0049
13	1	1	0	1	0	0.0019	0.0085	0.0044
14	1	0	1	1	0	0.0017	0.0103	0.0069
15	0	1	1	1	0	0.0035	0.0167	0.0081
16	1	1	1	1	0	0.0035	0.0167	0.0081

Note: "1" means that a 0.1 degree of belief is reassigned and move toward the maximal decrement of risk of industrial safety of each district.

**Table 7:** True Risk Inference (*TRI*).

Row	I	II	III	IV	*TRI* OF RISK OF DISTRICT			
					A	B	C	D
1	0	0	0	0	0	0	0	0
2	1	0	0	0	0.00125	0.0021	0.00365	0.00245
3	0	1	0	0	0.00105	0.002	0.00315	0.00275
4	0	0	1	0	0.00135	0.0031	0.00535	0.00295
5	0	0	0	1	0.00105	0.0017	0.00445	0.0024
6	1	1	0	0	0.0024	0.0042	0.0068	0.00525
7	1	0	1	0	0.00275	0.00535	0.00895	0.0055
8	1	0	0	1	0.0024	0.00395	0.00795	0.00495
9	0	1	1	0	0.00245	0.00515	0.00855	0.00575
10	0	1	0	1	0.00215	0.0038	0.00765	0.0052
11	0	0	1	1	0.00245	0.0029	0.00685	0.0034
12	1	1	1	0	0.00395	0.0075	0.01215	0.00835
13	1	1	0	1	0.0036	0.0061	0.01115	0.0078
14	1	0	1	1	0.00395	0.00735	0.0133	0.00805
15	0	1	1	1	0.0037	0.0071	0.01305	0.0083
16	1	1	1	1	0.0053	0.00955	0.0165	0.011

*Note*: I stands for number of major hazard sources; II stands for number of hidden dangers discovered; III stands for number of units with harm of occupational disease; and IV stands for number of people contacted with occupational disease.

## 5. Conclusion

This paper proposes a new RIS assessment method using the hybrid of ER and AHP. A hierarchical structure of indexes to evaluate the comprehensive risk of RIS is constructed, which can be used as the reference to guide the development of RIS assessment models for other metropolitan cities. Compared to the real data from Beijing Work Safety Statistical Yearbook, the evaluation results of this model reflect the reality to a very high extent. For instance, in terms of the accidents, district C is of the highest risk value, and district A, L, N possess low risk values, which is in line with the reality reflected by historical data. However, the model can take into account both qualitative and quantitative data, which is more all-sided, and deal with the associated uncertainty to realise comprehensive RIS assessment against different variables and thus, aid to know the overall safety performance of different districts, which would not be achieved from the statistical analysis alone.

The contribution of this paper is made by the findings on the comparison of RIS risk levels of different regions against various risk factors so that best practices from the good performer(s) can be used to improve the safety of the others. The evaluation results can provide suggestive, useful and scientific support for the governments to rationally allocate the industrial safety resources to make metropolitan cities safer.

## Supporting information

S1 Table Initial data. (DOCX)

S2 Table Normalized data. (DOCX)

S3 Table Fuzzy belief structure. (DOCX)

S1 Questionnaire Questionnaire in English. (DOCX)

S2 Questionnaire Data. (DOCX)
