# Supplementary material for: Use of evidential reasoning and AHP to assess regional industrial safety
Source: PLoS One. 2018 May 24;13(5):e0197125. doi: 10.1371/journal.pone.0197125 (PMC5993124; doi:10.1371/journal.pone.0197125)
Supplement: S1 Table — (DOCX) [file pone.0197125.s001.docx]

**Supporting information**

S1 Table. Initial Data

| **level 1** | **level 2** | **level 3** | **level 4** | **A** | **B** | **C** | **D** | **E** | **F** | **G** | **H** | **I** | **G** | **K** | **L** | **M** | **N** | **O** | **P** | Data Source | |
| --- | --- | --- | --- | --- | --- | --- | --- | --- | --- | --- | --- | --- | --- | --- | --- | --- | --- | --- | --- | --- | --- |
| disaster-inducing factors | accidents | severity | death toll of industrial safety issues | 17 | 17 | 189 | 90 | 68 | 18 | 112 | 92 | 113 | 113 | 50 | 14 | 32 | 28 | 38 | 29 | Beijing work safety statistical yearbook 2014 | Page 7 |
|  |  |  | frequency of industrial safety issues | 12 | 17 | 168 | 84 | 63 | 16 | 105 | 80 | 101 | 100 | 43 | 14 | 32 | 24 | 37 | 29 | Beijing work safety statistical yearbook 2014 | Page 38 |
|  |  | accountability | number of people investigated and affixed liability | 0 | 0 | 4 | 22 | 5 | 1 | 0 | 0 | 0 | 1 | 3 | 1 | 3 | 0 | 1 | 0 | Beijing work safety statistical yearbook 2013 | Page 34 |
|  |  |  | the fines of ISA | 157.8 | 261.4 | 1077.23 | 356.61 | 541.45 | 254.15 | 128.87 | 328.25 | 375.3 | 124.1 | 40.94 | 65.4 | 74.97 | 43.1 | 73 | 600.01 | Beijing work safety statistical yearbook 2013 | Page 40 |
|  | hidden dangers | number of major hazard sources | | 0 | 0 | 4 | 3 | 12 | 3 | 98 | 13 | 16 | 10 | 9 | 0 | 0 | 0 | 1 | 0 | statistical data from Beijing Administration of Work Safety |  |
|  |  | number of hidden dangers discovered | | 8412 | 34708 | 99847 | 16636 | 33652 | 17088 | 6457 | 9969 | 10126 | 30082 | 27389 | 3861 | 11866 | 9165 | 14087 | 5342 | Beijing work safety statistical yearbook 2013 | Page 40 |
|  |  | number of units with harm of occupational disease | | 31 | 118 | 620 | 340 | 279 | 109 | 680 | 694 | 687 | 339 | 613 | 112 | 355 | 292 | 405 | 141 | Beijing work safety statistical yearbook 2013 | Page 53 |
|  |  | number of people contacted with occupational disease | | 1780 | 3826 | 5747 | 17424 | 10823 | 5371 | 14098 | 21869 | 27434 | 2567 | 11946 | 1268 | 8641 | 8700 | 6120 | 2959 | Beijing work safety statistical yearbook 2013 | Page 55 |
| vulnerability of hazard-affected carriers | vulnerability | population vulnerability | the resident population density | 21715 | 25787 | 8440 | 8302 | 7394 | 7638 | 508 | 1463 | 964 | 1406 | 1454 | 209 | 180 | 444 | 214 | 158 | Beijing Statistical Information Net | http://www.bjstats.gov.cn/ |
|  |  |  | proportion of aged population | 0.1287 | 0.1412 | 0.0911 | 0.0741 | 0.0973 | 0.1025 | 0.101 | 0.086 | 0.0773 | 0.0746 | 0.0703 | 0.1221 | 0.0969 | 0.1114 | 0.105 | 0.1044 | Beijing Statistical Information Net | http://www.bjstats.gov.cn/ |
|  |  |  | proportion of children | 0.0803 | 0.0867 | 0.0935 | 0.0886 | 0.0973 | 0.0885 | 0.1099 | 0.092 | 0.0956 | 0.0953 | 0.1009 | 0.1023 | 0.1126 | 0.1019 | 0.1071 | 0.1108 | Beijing Statistical Information Net | http://www.bjstats.gov.cn/ |
|  |  | infrastructural vulnerability | number of gas station per km^2^ | 0.287 | 0.336 | 0.297 | 0.181 | 0.294 | 0.190 | 0.070 | 0.104 | 0.085 | 0.056 | 0.096 | 0.010 | 0.012 | 0.038 | 0.017 | 0.019 | Beijing Municipal Commission of City Management | www.bjmac.gov.cn/csyxbz/ |
|  |  | economical vulnerability | the reciprocal of regional GDP per capita | 5.79E-06 | 4.61E-06 | 9.69E-06 | 9.32E-06 | 2.24E-05 | 1.76E-05 | 2.10E-05 | 2.65E-05 | 7.98E-06 | 3.39E-05 | 3.49E-05 | 2.44E-05 | 1.91E-05 | 2.50E-05 | 2.44E-05 | 3.43E-05 | Beijing Statistical Information Net | http://www.bjstats.gov.cn/ |
|  |  |  | unemployment rate | 0.005 | 0.004 | 0.002 | 0.002 | 0.004 | 0.006 | 0.006 | 0.003 | 0.003 | 0.002 | 0.002 | 0.013 | 0.006 | 0.005 | 0.004 | 0.007 | Beijing Statistical Information Net | http://www.bjstats.gov.cn/ |
|  | adaptability | employee's assurance | (-)number of employees joined medical assurance | 1190326 | 1577177 | 2294871 | 2115309 | 659095 | 281588 | 262332 | 421955 | 469264 | 344709 | 403052 | 142918 | 169117 | 149800 | 158206 | 105222 | Beijing Statistical Information Net | http://www.bjstats.gov.cn/ |
|  |  |  | (-)number of employees joined unemployment insurance | 1025641 | 1404440 | 2189639 | 2000938 | 575847 | 244228 | 246187 | 307271 | 421580 | 316707 | 346894 | 138066 | 158113 | 129756 | 135567 | 61168 | Beijing Statistical Information Net | http://www.bjstats.gov.cn/ |
|  |  | protection | (-)investment of infrastructure | 87.81 | 50.97 | 371.98 | 155.29 | 197.63 | 62.45 | 146.62 | 134.64 | 94.03 | 113.75 | 93.22 | 63.35 | 42.28 | 29.71 | 77.28 | 37.46 | Beijing Statistical Information Net | http://www.bjstats.gov.cn/ |
|  |  |  | (-)number of medical staff per thousand people | 20.41 | 18.82 | 8.72 | 6.17 | 5.68 | 8.64 | 6.37 | 4.23 | 4.96 | 4.18 | 4.7 | 8.29 | 6.27 | 6.68 | 5.61 | 5.34 | Beijing Statistical Information Net | http://www.bjstats.gov.cn/ |
|  |  |  | (-)number of hospital beds per thousand people | 11.87 | 11.13 | 4.6 | 2.68 | 3.83 | 5.47 | 5.14 | 1.83 | 2.52 | 4.75 | 3.57 | 7.92 | 3.58 | 4.11 | 2.37 | 2.39 | Beijing Statistical Information Net | http://www.bjstats.gov.cn/ |
| safety control | supervision | regulatory capacity | (-)coverage rate of supervision | 0.0661 | 0.137 | 0.6754 | 0.124 | 0.0822 | 0.1557 | 0.2615 | 0.2094 | 0.3267 | 0.2601 | 0.6849 | 0.3747 | 0.3451 | 0.2935 | 0.2701 | 0.1199 | Beijing work safety statistical yearbook 2013 | Page 46 |
|  |  |  | (-)economic punishment | 40.14 | 124.5 | 951.15 | 362.89 | 192.7 | 53.3 | 164 | 422.5 | 278.35 | 76.62 | 385.85 | 162.22 | 56.3 | 47.7 | 40 | 96.69 | Beijing work safety statistical yearbook 2013 | Page 47 |
|  |  |  | (-)punishment rate of supervision | 0.0377 | 0.0729 | 0.0332 | 0.0422 | 0.0478 | 0.0351 | 0.0508 | 0.0831 | 0.1323 | 0.0352 | 0.0277 | 0.0361 | 0.0446 | 0.0313 | 0.0278 | 0.04 | Beijing work safety statistical yearbook 2013 | Page 47 |
|  |  | personnel allocation | (-)crew size of safety supervision system | 52 | 67 | 66 | 54 | 57 | 33 | 77 | 55 | 49 | 83 | 75 | 46 | 43 | 37 | 45 | 43 | Beijing work safety statistical yearbook 2013 | Page 67 |
|  |  |  | (-)number of people attending the inspection | 12649 | 45335 | 58822 | 14422 | 43494 | 21378 | 18684 | 6144 | 11214 | 33466 | 46571 | 12529 | 15000 | 8212 | 14456 | 11291 | Beijing work safety statistical yearbook 2013 | Page 40 |
|  |  |  | (-)*capacity of the safety supervision crew | (0,0,0.1,0.4,0.5) | (0,0,0.1,0.6,0.3) | (0,0,0.2,0.4,0.4) | (0,0.1,0.1,0.5,0.3) | (0,0,0.2,0.6,0.2) | (0,0.1,0.3,0.5,0.1) | (0,0,0.3,0.6,0.1) | (0,0.1,0.1,0.4,0.4) | (0,0.2,0.6,0.2） | (0,0.1,0.3,0.4,0.2) | (0,0.1,0.3,0.4,0.2) | (0,0.1,0.3,0.5,0.1) | (0,0.1,0.2,0.5,0.2) | (0,0.1,0.3,0.5,0.1) | (0,0.1,0.2,0.6,0.1) | (0,0.1,0.3,0.5,0.1) | statistical data from questionnaire |  |
|  | emergency management & publicity | emergency capacity | (-)number of fire brigade | 9 | 9 | 21 | 16 | 12 | 5 | 4 | 6 | 9 | 8 | 11 | 3 | 5 | 4 | 3 | 6 | [statistical data from website](file:///D:\czc\硕士\【9】数据相关\北京市\安全生产\数据整理\消防数据1.jpg) |  |
|  |  |  | (-)emergency resources reserves | 44.714 | 51.052 | 50.87 | 41.818 | 45.078 | 33.13 | 33.494 | 42.182 | 44.714 | 32.402 | 54.494 | 26.974 | 32.584 | 26.974 | 29.688 | 14.662 | statistical data from Beijing Administration of Work Safety |  |
|  |  | safety propaganda | (-)number of news manuscripts about industrial safety | 170 | 429 | 829 | 788 | 634 | 170 | 69 | 245 | 645 | 1066 | 95 | 465 | 677 | 934 | 895 | 275 | Beijing work safety statistical yearbook 2013 | Page 66 |
|  |  |  | (-)*the level of public safety awareness | (0,0.1,0.1,0.4,0.4) | (0,0,0.2,0.4,0.4) | (0,0,0.2,0.6,0.2) | (0,0,0.2,0.5,0.3) | (0.1,0,0.4,0.5,0) | (0,0.1,0.2,0.6,0.1) | (0,0.1,0.3,0.5,0.1) | (0,0.1,0.2,0.6,0.1) | (0,0.1,0.4,0,4,0.1) | (0.1,0,0.4,0.5,0) | (0.1,0.1,0.4,0.4,0) | (0.1,0,0.5,0.4,0) | (0.1,0,0.5,0.4,0) | (0.1,0,0.5,0.4,0) | (0.1,0,0.5,0.4,0) | (0.1,0,0.4,0.5,0) | statistical data from questionnaire |  |

* A-P in the table S1 , table S2, table S3 and other places in this paper stand for the 16 districts including Dongcheng, Xicheng, Shijingshan, Chaoyang, Fengtai, Fangshan, Haidian, Tongzhou, Shunyi, Daxing, Changping, Mentougou, Pinggu, Huairou, Miyun, and Yanqing, but not respectively.
