# Supplementary material for: Use of evidential reasoning and AHP to assess regional industrial safety
Source: PLoS One. 2018 May 24;13(5):e0197125. doi: 10.1371/journal.pone.0197125 (PMC5993124; doi:10.1371/journal.pone.0197125)
Supplement: S2 Table — (DOCX) [file pone.0197125.s002.docx]

**Supporting information**

S2 Table. Normalized Data

| level 1 | level 2 | level 3 | level 4 | A | B | C | D | E | F | G | H | I | J | K | L | M | N | O | P |
| --- | --- | --- | --- | --- | --- | --- | --- | --- | --- | --- | --- | --- | --- | --- | --- | --- | --- | --- | --- |
| disaster-inducing factors | accidents | severity | death toll of industrial safety issues | 0.0171 | 0.0171 | 1 | 0.4343 | 0.3086 | 0.0229 | 0.56 | 0.4457 | 0.5657 | 0.5657 | 0.2057 | 0 | 0.1029 | 0.08 | 0.1371 | 0.0857 |
|  |  |  | frequency of industrial safety issues | 0 | 0.0321 | 1 | 0.4615 | 0.3269 | 0.0256 | 0.5962 | 0.4359 | 0.5705 | 0.5641 | 0.1987 | 0.0128 | 0.1282 | 0.0769 | 0.1603 | 0.1090 |
|  |  | accountability | number of people investigated and affixed liability | 0 | 0 | 0.1818 | 1 | 0.2273 | 0.0455 | 0 | 0 | 0 | 0.0455 | 0.1364 | 0.0455 | 0.1364 | 0 | 0.0455 | 0 |
|  |  |  | the fines of ISA | 0.1128 | 0.2127 | 1 | 0.3046 | 0.4830 | 0.2057 | 0.0849 | 0.2772 | 0.3227 | 0.0802 | 0 | 0.0236 | 0.0328 | 0.0021 | 0.0309 | 0.5395 |
|  | hidden dangers | number of major hazard sources | | 0 | 0 | 0 | 0.0408 | 0.0306 | 0.1224 | 0.0306 | 1 | 0.1327 | 0.1633 | 0.1020 | 0.0918 | 0 | 0 | 0 | 0.01022 |
|  |  | number of hidden dangers discovered | | 0.0474 | 0.0474 | 0.3214 | 1 | 0.1331 | 0.3104 | 0.1378 | 0.0270 | 0.0636 | 0.0653 | 0.2732 | 0.2451 | 0 | 0.0834 | 0.0553 | 0.1065 |
|  |  | number of units with harm of occupational disease | | 0 | 0 | 0.1312 | 0.8884 | 0.4661 | 0.3741 | 0.1176 | 0.9789 | 1 | 0.9894 | 0.4646 | 0.8778 | 0.1222 | 0.4887 | 0.3937 | 0.5641 |
|  |  | number of people contacted with occupational disease | | 0.0196 | 0.0196 | 0.0978 | 0.1712 | 0.6174 | 0.3652 | 0.1568 | 0.4903 | 0.7873 | 1 | 0.0496 | 0.4081 | 0 | 0.2818 | 0.2840 | 0.1854 |
| vulnerability of hazard-affected carriers | vulnerability | population vulnerability | the resident population density | 0.8411 | 1 | 0.3231 | 0.3178 | 0.2823 | 0.2919 | 0.0137 | 0.0509 | 0.0314 | 0.0487 | 0.0506 | 0.0020 | 0.0009 | 0.0112 | 0.0022 | 0 |
|  |  |  | proportion of aged population | 0.8237 | 1 | 0.2934 | 0.0536 | 0.3808 | 0.4542 | 0.4330 | 0.2214 | 0.0987 | 0.0606 | 0 | 0.7306 | 0.3752 | 0.5797 | 0.4894 | 0.4810 |
|  |  |  | proportion of children | 0 | 0.1981 | 0.4087 | 0.2570 | 0.5263 | 0.2539 | 0.9164 | 0.3622 | 0.4737 | 0.4644 | 0.6378 | 0.6811 | 1 | 0.6687 | 0.8297 | 0.9443 |
|  |  | infrastructural vulnerability | number of gas station per km^2^ | 0.8474 | 1 | 0.8780 | 0.5236 | 0.8708 | 0.5502 | 0.1825 | 0.2864 | 0.2299 | 0.1395 | 0.2612 | 0 | 0.0059 | 0.0845 | 0.0206 | 0.0252 |
|  |  | economical vulnerability | the reciprocal of regional GDP per capita | 0.0388 | 0 | 0.1676 | 0.1555 | 0.5882 | 0.4298 | 0.5396 | 0.7225 | 0.1111 | 0.9665 | 1 | 0.6529 | 0.4768 | 0.6731 | 0.6527 | 0.9783 |
|  |  |  | unemployment rate | 0.2583 | 0.2376 | 0.0094 | 0.0359 | 0.1753 | 0.4056 | 0.4177 | 0.0925 | 0.0749 | 0 | 0.0285 | 1 | 0.3380 | 0.2767 | 0.1688 | 0.4358 |
|  | adaptability | employee's assurance | number of employees joined medical assurance | 0.5044 | 0.3278 | 0 | 0.0820 | 0.7470 | 0.9195 | 0.9282 | 0.8553 | 0.8337 | 0.8906 | 0.8640 | 0.9828 | 0.9708 | 0.9796 | 0.9758 | 1 |
|  |  |  | number of employees joined unemployment insurance | 0.5469 | 0.3689 | 0 | 0.0887 | 0.7582 | 0.9140 | 0.9131 | 0.8844 | 0.8307 | 0.8800 | 0.8658 | 0.9639 | 0.9545 | 0.9678 | 0.9650 | 1 |
|  |  | protection | investment of infrastructure | 0.8303 | 0.9379 | 0 | 0.6331 | 0.5094 | 0.9043 | 0.6584 | 0.6934 | 0.8121 | 0.7545 | 0.8144 | 0.9017 | 0.9633 | 1 | 0.8610 | 0.9774 |
|  |  |  | number of medical staff per thousand people | 0 | 0.0980 | 0.7200 | 0.8774 | 0.9076 | 0.7252 | 0.8651 | 0.9969 | 0.9519 | 1 | 0.9680 | 0.7468 | 0.8712 | 0.8460 | 0.9119 | 0.9285 |
|  |  |  | number of hospital beds per thousand people | 0 | 0.0737 | 0.7241 | 0.9153 | 0.8008 | 0.6375 | 0.6703 | 1 | 0.9313 | 0.7092 | 0.8267 | 0.3934 | 0.8257 | 0.7729 | 0.9462 | 0.9442 |
| safety control | supervision | regulatory capacity | coverage rate of supervision | 1 | 0.8854 | 0.0154 | 0.9064 | 0.9740 | 0.8552 | 0.6842 | 0.7684 | 0.5789 | 0.6865 | 0 | 0.5013 | 0.5491 | 0.6325 | 0.6703 | 0.9131 |
|  |  |  | economic punishment | 0.9998 | 0.9073 | 0 | 0.6456 | 0.8324 | 0.9854 | 0.8639 | 0.5802 | 0.7384 | 0.9598 | 0.6204 | 0.8659 | 0.9821 | 0.9915 | 1 | 0.9378 |
|  |  |  | punishment rate of supervision | 0.9044 | 0.5679 | 0.9474 | 0.8614 | 0.8078 | 0.9293 | 0.779 | 0.4704 | 0 | 0.9283 | 1 | 0.9197 | 0.8384 | 0.9656 | 0.9990 | 0.8824 |
|  |  | personnel allocation | crew size of security supervision system | 0.62 | 0.32 | 0.34 | 0.58 | 0.52 | 1 | 0.12 | 0.56 | 0.68 | 0 | 0.16 | 0.74 | 0.8 | 0.92 | 0.76 | 0.8 |
|  |  |  | number of people attending the inspection | 0.8765 | 0.2560 | 0 | 0.8429 | 0.2910 | 0.7108 | 0.7619 | 1 | 0.9038 | 0.4813 | 0.2326 | 0.8788 | 0.8319 | 0.9607 | 0.8422 | 0.9023 |
|  |  |  | *capacity of the safety supervision crew | (0,0.7,0.3,0,0) | (0,0.5,0.5,0,0) | (0.2,0.8,0,0,0) | (0,0.2,0.8,0,0) | (0,0.4,0.6,0,0) | (0,0,1,0,0) | (0,0,0.8,0.2,0) | (0,0.3,0.7,0,0) | (0,0.6,0.4,0,0) | (0,0.5,0.5,0,0) | (0,0,0.8,0.2,0) | (0,0,0.7,0.3,0) | (0,0,0,0.8,0.2) | (0,0,0.5,0.5,0) | (0,0,0.7,0.3,0) | (0,0,0,0.9,0.1) |
|  | emergency management & publicity | emergency capacity | number of fire brigade | 0.6667 | 0.6667 | 0 | 0.2778 | 0.5 | 0.8889 | 0.9444 | 0.8333 | 0.6667 | 0.7222 | 0.5556 | 1 | 0.8889 | 0.9444 | 1 | 0.8333 |
|  |  |  | emergency resources reserves | 0.2455 | 0.0864 | 0.0910 | 0.3182 | 0.2364 | 0.5364 | 0.5272 | 0.3091 | 0.2455 | 0.5546 | 0 | 0.6909 | 0.5501 | 0.6909 | 0.6228 | 1 |
|  |  | safety propaganda | number of news manuscripts about industrial safety | 0.8987 | 0.6389 | 0.2377 | 0.2788 | 0.4333 | 0.8987 | 1 | 0.8235 | 0.4223 | 0 | 0.9739 | 0.6028 | 0.3902 | 0.1324 | 0.1715 | 0.7934 |
|  |  |  | *the level of public safety awareness | (0,0.8,0.2,0,0) | (0,0.7,0.3,0,0) | (0,0.9,0.1,0,0) | (0,0.6,0.4,0,0) | (0,0.5,0.5,0,0) | (0,0.4,0.6,0,0) | (0,0.5,0.5,0,0) | (0,0.7,0.3,0,0) | (0,0.5,0.5,0,0) | (0,0.8,0.2,0,0) | (0,0.3,0.7,0,0) | (0,0.4,0.6,0,0) | (0,0.5,0.5,0,0) | (0,0,1,0,0) | (0,0.5,0.5,0,0) | (0,0.4,0.6,0,0) |
