# Supplementary material for: Use of evidential reasoning and AHP to assess regional industrial safety
Source: PLoS One. 2018 May 24;13(5):e0197125. doi: 10.1371/journal.pone.0197125 (PMC5993124; doi:10.1371/journal.pone.0197125)
Supplement: S3 Table — (DOCX) [file pone.0197125.s003.docx]

**Supporting information**

S3 Table. Fuzzy Belief Structure

| lvl 1 | lvl 2 | lvl 3 | level 4 | A | B | C | D | E | F | G | H | I | J | K | L | M | N | O | P |
| --- | --- | --- | --- | --- | --- | --- | --- | --- | --- | --- | --- | --- | --- | --- | --- | --- | --- | --- | --- |
| disaster-inducing factors | accidents | *S* | death toll of industrial safety issues | (1,0,0,0,0) | (1,0,0,0,0) | (0,0,0,0,1) | (0,0,1,0,0) | (0,0.91,0.09,0,0) | (1,0,0,0,0) | (0,0,1,0,0) | (0,0,1,0,0) | (0,0,1,0,0) | (0,0,1,0,0) | (0,1,0,0,0) | (1,0,0,0,0) | (0.94,0.06,0,0,0) | (1,0,0,0,0) | (0.46,0.54,0,0,0) | (1,0,0,0,0) |
|  |  |  | frequency of industrial safety issues | (1,0,0,0,0) | (1,0,0,0,0) | (0,0,0,0,1) | (0,0,1,0,0) | (0,0.73,0.27,0,0) | (1,0,0,0,0) | (0,0,1,0,0) | (0,0,1,0,0) | (0,0,1,0,0) | (0,0,1,0,0) | (0.01,0.99,0,0,0) | (1,0,0,0,0) | (0.56,0.44,0,0,0) | (1,0,0,0,0) | (0.25,0.75,0,0,0) | (0.84,0.16,0,0,0) |
|  |  | *A* | number of people investigated and affixed liability | (1,0,0,0,0) | (1,0,0,0,0) | (0.1,0.9,0,0,0) | (0,0,0,0,1) | (0,1,0,0,0) | (1,0,0,0,0) | (1,0,0,0,0) | (1,0,0,0,0) | (1,0,0,0,0) | (1,0,0,0,0) | (0.47,0.53,0,0,0) | (1,0,0,0,0) | (0.47,0.53,0,0,0) | (1,0,0,0,0) | (1,0,0,0,0) | (1,0,0,0,0) |
|  |  |  | the fines | (0.77,0.23,0,0,0) | (0,1,0,0,0) | (0,0,0,0,1) | (0,0.95,0.05,0,0) | (0,0,1,0,0) | (0,1,0,0,0) | (1,0,0,0,0) | (0,1,0,0,0) | (0,0.77,0.23,0,0) | (1,0,0,0,0) | (1,0,0,0,0) | (1,0,0,0,0) | (1,0,0,0,0) | (1,0,0,0,0) | (1,0,0,0,0) | (0,0,1,0,0) |
|  | hidden dangers | number of major hazard sources | | (1,0,0,0,0) | (1,0,0,0,0) | (1,0,0,0,0) | (1,0,0,0,0) | (1,0,0,0,0) | (0.63,0.37,0,0,0) | (1,0,0,0,0) | (0,0,0,0,1) | (0.51,0.49,0,0,0) | (0.23,0.77,0,0,0) | (0.96,0.04,0,0,0) | (1,0,0,0,0) | (1,0,0,0,0) | (1,0,0,0,0) | (1,0,0,0,0) | (1,0,0,0,0) |
|  |  | number of hidden dangers discovered | | (1,0,0,0,0) | (1,0,0,0,0) | (0,0.79,0.21,0,0) | (0,0,0,0,1) | (0.5,0.5,0,0,0) | (0,0.9,0.1,0,0) | (0.45,0.55,0,0,0) | (1,0,0,0,0) | (1,0,0,0,0) | (1,0,0,0,0) | (0,1,0,0,0) | (0,1,0,0,0) | (1,0,0,0,0) | (1,0,0,0,0) | (1,0,0,0,0) | (0.88,0.12,0,0,0) |
|  |  | number of units with harm of occupational disease | | (1,0,0,0,0) | (1,0,0,0,0) | (0.52,0.48,0,0,0) | (0,0,0,0.21,0.79) | (0,0,1,0,0) | (0,0.26,0.74,0,0) | (0.7,0.3,0,0,0) | (0,0,0,0,1) | (0,0,0,0,1) | (0,0,0,0,1) | (0,0,1,0,0) | (0,0,0,0.36,0.64) | (0.64,0.36,0,0,0) | (0,0,1,0,0) | (0,0.06,0.94,0,0) | (0,0,1,0,0) |
|  |  | number of people contacted with occupational disease | | (1,0,0,0,0) | (1,0,0,0,0) | (1,0,0,0,0) | (0.17,0.83,0,0,0) | (0,0,0.83,0.17,0) | (0,0.35,0.65,0,0) | (0.28,0.72,0,0,0) | (0,0,1,0,0) | (0,0,0,1,0) | (0,0,0,0,1) | (1,0,0,0,0) | (0,0,1,0,0) | (1,0,0,0,0) | (0,1,0,0,0) | (0,1,0,0,0) | (0.08,0.92,0,0,0) |
| vulnerability of hazard-affected carriers | vulnerability | *PV* | the resident population densit | (0,0,0,0.74,0.26) | (0,0,0,0,1) | (0,0.77,0.23,0,0) | (0,0.82,0.18,0,0) | (0,1,0,0,0) | (0,1,0,0,0) | (1,0,0,0,0) | (1,0,0,0,0) | (1,0,0,0,0) | (1,0,0,0,0) | (1,0,0,0,0) | (1,0,0,0,0) | (1,0,0,0,0) | (1,0,0,0,0) | (1,0,0,0,0) | (1,0,0,0,0) |
|  |  |  | proportion of aged population | (0,0,0,0.87,0.13) | (0,0,0,0,1) | (0,1,0,0,0) | (1,0,0,0,0) | (0,0.19,0.81,0,0) | (0,0,1,0,0) | (0,0,1,0,0) | (0,1,0,0,0) | (1,0,0,0,0) | (1,0,0,0,0) | (1,0,0,0,0) | (0,0,0,1,0) | (0,0.25,0.75,0,0) | (0,0,1,0,0) | (0,0,1,0,0) | (0,0,1,0,0) |
|  |  |  | proportion of children | (1,0,0,0,0) | (0.01,0.99,0,0,0) | (0,0,1,0,0) | (0,1,0,0,0) | (0,0,1,0,0) | (0,1,0,0,0) | (0,0,0,0,1) | (0,0.38,0.62,0,0) | (0,0,1,0,0) | (0,0,1,0,0) | (0,0,0.62,0.38,0) | (0,0,0.19,0.81,0) | (0,0,0,0,1) | (0,0,0.31,0.69,0) | (0,0,0,0.83,0.17) | (0,0,0,0,1) |
|  |  | *IV* | number of gas station per km^2^ | (0,0,0,0.69,0.31) | (0,0,0,0,1) | (0,0,0,0.36,0.64) | (0,0,1,0,0) | (0,0,0,0.45,0.55) | (0,0,1,0,0) | (0.1,0.9,0,0,0) | (0,1,0,0,0) | (0,1,0,0,0) | (0.43,0.57,0,0,0) | (0,1,0,0,0) | (1,0,0,0,0) | (1,0,0,0,0) | (1,0,0,0,0) | (1,0,0,0,0) | (1,0,0,0,0) |
|  |  | *EV* | the reciprocal of regional GDP per capita | (1,0,0,0,0) | (1,0,0,0,0) | (0.19,0.81,0,0,0) | (0.29,0.71,0,0,0) | (0,0,1,0,0) | (0,0,1,0,0) | (0,0,1,0,0) | (0,0,0,1,0) | (0.8,0.2,0,0,0) | (0,0,0,0,1) | (0,0,0,0,1) | (0,0,0.47,0.53,0) | (0,0,1,0,0) | (0,0,0.27,0.73,0) | (0,0,0.47,0.53,0) | (0,0,0,0,1) |
|  |  |  | unemployment rate | (0,1,0,0,0) | (0,1,0,0,0) | (1,0,0,0,0) | (1,0,0,0,0) | (0.14,0.86,0,0,0) | (0,0,1,0,0) | (0,0,1,0,0) | (1,0,0,0,0) | (1,0,0,0,0) | (1,0,0,0,0) | (1,0,0,0,0) | (0,0,0,0,1) | (0,0.62,0.38,0,0) | (0,1,0,0,0) | (0.18,0.82,0,0,0) | (0,0,1,0,0) |
|  | adaptability | *EA* | number of employees joined medical assurance | (0,0,1,0,0) | (0,0.72,0.28,0,0) | (1,0,0,0,0) | (1,0,0,0,0) | (0,0,0,1,0) | (0,0,0,0,1) | (0,0,0,0,1) | (0,0,0,0.62,0.38) | (0,0,0,0.8,0.2) | (0,0,0,0.17,0.83) | (0,0,0,0.53,0.47) | (0,0,0,0,1) | (0,0,0,0,1) | (0,0,0,0,1) | (0,0,0,0,1) | (0,0,0,0,1) |
|  |  |  | number of employees joined unemployment insurance | (0,0,1,0,0) | (0,0.31,0.69,0,0) | (1,0,0,0,0) | (1,0,0,0,0) | (0,0,0,1,0) | (0,0,0,0,1) | (0,0,0,0,1) | (0,0,0,0.27,0.73) | (0,0,0,0.82,0.18) | (0,0,0,0.33,0.67) | (0,0,0,0.51,0.49) | (0,0,0,0,1) | (0,0,0,0,1) | (0,0,0,0,1) | (0,0,0,0,1) | (0,0,0,0,1) |
|  |  | *P* | investment of infrastructure 0.3333 | (0,0,0,0.82,0.18) | (0,0,0,0,1) | (1,0,0,0,0) | (0,0,0.67,0.33,0) | (0,0,1,0,0) | (0,0,0,0,1) | (0,0,0.42,0.58,0) | (0,0,0.07,0.93,0) | (0,0,0,0.94,0.06) | (0,0,0,1,0) | (0,0,0,0.92,0.08) | (0,0,0,0,1) | (0,0,0,0,1) | (0,0,0,0,1) | (0,0,0,0.56,0.44) | (0,0,0,0,1) |
|  |  |  | number of medical staff per thousand people | (1,0,0,0,0) | (1,0,0,0,0) | (0,0,0,1,0) | (0,0,0,0.37,0.63) | (0,0,0,0,1) | (0,0,0,1,0) | (0,0,0,0.52,0.48) | (0,0,0,0,1) | (0,0,0,0,1) | (0,0,0,0,1) | (0,0,0,0,1) | (0,0,0,1,0) | (0,0,0,0.45,0.55) | (0,0,0,0.7,0.3) | (0,0,0,0,1) | (0,0,0,0,1) |
|  |  |  | number of hospital beds per thousand people | (1,0,0,0,0) | (1,0,0,0,0) | (0,0,0,1,0) | (0,0,0,0,1) | (0,0,0,1,0) | (0,0,0.63,0.37,0) | (0,0,0.3,0.7,0) | (0,0,0,0,1) | (0,0,0,0,1) | (0,0,0,1,0) | (0,0,0,0.85,0.15) | (0,0.07,0.93,0,0) | (0,0,0,0.85,0.15) | (0,0,0,1,0) | (0,0,0,0,1) | (0,0,0,0,1) |
| safety control | supervision | *RC* | coverage rate of supervision | (0,0,0,0,1) | (0,0,0,0.25,0.75) | (1,0,0,0,0) | (0,0,0,0,1) | (0,0,0,0,1) | (0,0,0,0.62,0.38) | (0,0,0.16,0.84) | (0,0,0,1,0) | (0,0,1,0,0) | (0,0,0.14,0.86,0) | (1,0,0,0,0) | (0,0,1,0,0) | (0,0,1,0,0) | (0,0,0.67,0.33,0) | (0,0,0.3,0.7,0) | (0,0,0,0,1) |
|  |  |  | economic punishment | (0,0,0,0,1) | (0,0,0,0,1) | (1,0,0,0,0) | (0,0,0.54,0.46,0) | (0,0,0,0.81,0.19) | (0,0,0,0,1) | (0,0,0,0.53,0.47) | (0,0,1,0,0) | (0,0,0,1,0) | (0,0,0,0,1) | (0,0,0.8,0.2,0) | (0,0,0,0.51,0.49) | (0,0,0,0,1) | (0,0,0,0,1) | (0,0,0,0,1) | (0,0,0,0,1) |
|  |  |  | punishment rate of supervision | (0,0,0,0,1) | (0,0,1,0,0) | (0,0,0,0,1) | (0,0,0,0.56,0.44) | (0,0,0,0.96,0.04) | (0,0,0,0,1) | (0,0,0,1,0) | (0,0,1,0,0) | (1,0,0,0,0) | (0,0,0,0,1) | (0,0,0,0,1) | (0,0,0,0,1) | (0,0,0,0.76,0.24) | (0,0,0,0,1) | (0,0,0,0,1) | (0,0,0,0.3,0.7) |
|  |  | *PA* | crew size of safety supervision system | (0,0,0.8,0.2,0) | (0,0.8,0.2,0,0) | (0,0.6,0.4,0,0) | (0,0,1,0,0) | (0,0,1,0,0) | (0,0,0,0,1) | (0.67,0.33,0,0,0) | (0,0,1,0,0) | (0,0,0.2,0.8,0) | (1,0,0,0,0) | (0.25,0.75,0,0,0) | (0,0,0,1,0) | (0,0,0,1,0) | (0,0,0,0,1) | (0,0,0,1,0) | (0,0,0,1,0) |
|  |  |  | number of people attending the inspection | (0,0,0,0.38,0.62) | (0,1,0,0,0) | (1,0,0,0,0) | (0,0,0,0.73,0.27) | (0,1,0,0,0) | (0,0,0,1,0) | (0,0,0,1,0) | (0,0,0,0,1) | (0,0,0,0,1) | (0,0,1,0,0) | (0,1,0,0,0) | (0,0,0,0.35,0.65) | (0,0,0,0.81,0.19) | (0,0,0,0,1) | (0,0,0,0.73,0.27) | (0,0,0,0,1) |
|  |  |  | *capacity of the safety supervision crew | (0.5,0.4,0.1,0,0) | (0.3,0.6,0.1,0,0) | (0.4,0.4,0.2,0,0) | (0.3,0.5,0.1,0.1,0) | (0.2,0.6,0.2,0,0) | (0.1,0.5,0.3,0.1,0) | (0.1,0.6,0.3,0,0） | (0.4,0.4,0.1,0.1,0) | (0.2,0.6,0.2,0,0) | (0.2,0.4,0.3,0.1,0) | (0.2,0.4,0.3,0.1,0) | (0.1,0.5,0.3,0.1,0) | (0.2,0.5,0.2,0.1,0) | (0.1,0.5,0.3,0.1,0) | (0.1,0.6,0.2,0.1,0) | (0.1,0.5,0.3,0.1,0) |
|  | emergency management & publicity | *EC* | number of fire brigade | (0,0,0.33,0.67,0) | (0,0,0.33,0.67,0) | (1,0,0,0,0) | (0,1,0,0,0) | (0,0,1,0,0) | (0,0,0,0.2,0.8) | (0,0,0,0,1) | (0,0,0,0.8,0.2) | (0,0,0.33,0.67,0) | (0,0,0,1,0) | (0,0,1,0,0) | (0,0,0,0,1) | (0,0,0,0.2,0.8) | (0,0,0,0,1) | (0,0,0,0,1) | (0,0,0,0.8,0.2) |
|  |  |  | emergency resources reserves | (0,1,0,0,0) | (1,0,0,0,0) | (1,0,0,0,0) | (0,0.82,0.18,0,0) | (0,1,0,0,0) | (0,0,1,0,0) | (0,0,1,0,0) | (0,0.91,0.09,0,0) | (0,1,0,0,0) | (0,0,1,0,0) | (1,0,0,0,0) | (0,0,0.09,0.91,0) | (0,0,1,0,0) | (0,0,0.09,0.91,0) | (0,0,0.77,0.23,0) | (0,0,0,0,1) |
|  |  | *SP* | number of news manuscripts about industrial safety | (0,0,0,0.03,0.97) | (0,0,0.61,0.39,0) | (0,1,0,0,0) | (0,1,0,0,0) | (0,0,1,0,0) | (0,0,0,0.03,0.97) | (0,0,0,0,1) | (0,0,0,0.87,0.13) | (0,0,1,0,0) | (1,0,0,0,0) | (0,0,0,0,1) | (0,0,0.97,0.03,0) | (0,0.1,0.9,0,0) | (0.51,0.49,0,0,0) | (0.17,0.83,0,0,0) | (0,0,0,1,0) |
|  |  |  | *the level of public safety awareness | (0.4,0.4,0.1,0.1,0) | (0.4,0.4,0.2,0,0) | (0.2,0.6,0.2,0,0) | (0.3,0.5,0.2,0,0) | (0,0.5,0.4,0,0.1) | (0.1,0.6,0.2,0.1,0) | (0.1,0.5,0.3,0.1,0) | (0.1,0.6,0.2,0.1,0) | (0.1,0.4,0.4,0.1,0) | (0,0.5,0.4,0,0.1) | (0,0.4,0.4,0.1,0.1) | (0,0.4,0.5,0,0.1) | (0,0.4,0.5,0,0.1) | (0,0.4,0.5,0,0.1) | (0,0.4,0.5,0,0.1) | (0,0.5,0.4,0,0.1) |
