# Supplementary material for: Use of evidential reasoning and AHP to assess regional industrial safety
Source: PLoS One. 2018 May 24;13(5):e0197125. doi: 10.1371/journal.pone.0197125 (PMC5993124; doi:10.1371/journal.pone.0197125)
Supplement: S1 Questionnaire — (DOCX) [file pone.0197125.s004.docx]

**Supporting information**

S1 Questionnaire. Questionnaire in English

**Questionnaire of Indexes’ Weights and Qualitative Indexes’ data in the Assessment of Risk of Regional Industrial Safety (RIS) in Beijing**

Hello, this questionnaire is designed to assess the indexes’ weights in the assessment of risk of regional industrial safety in Beijing. Because of your rich experience of Beijing industrial safety, we invite you to take part in this questionnaire to give grades to assess the importance of each index in the index system. Please follow the guidance of this questionnaire and give your opinion of the grade of importance of each index. Many thanks for your help!

This questionnaire is made up by 3 parts, please do not leave out any one, thanks!

**Part 1 Overview of the index system**

Take the data availability and the situation of industrial safety of Beijing into consideration, we make the index system of assessing the risk of RIS of Beijing, as follows,

Table 1. **The Index System of Industrial Safety Comprehensive Risk**

| **level 1** | **level 2** | **level 3** | **level 4** |
| --- | --- | --- | --- |
| disaster-inducing factors | accidents | severity | death toll of industrial safety issues |
|  |  |  | frequency of industrial safety issues |
|  |  | accountability | number of people investigated and affixed liability |
|  |  |  | the fines of industrial safety accidents |
|  | hidden dangers | number of major hazard sources | |
|  |  | number of hidden dangers discovered | |
|  |  | number of units with harm of occupational disease | |
|  |  | number of people contacted with occupational disease | |
| vulnerability of hazard-affected carriers | vulnerability | population vulnerability | the resident population density |
|  |  |  | proportion of aged population |
|  |  |  | proportion of children |
|  |  | infrastructural vulnerability | number of gas station per km^2^ |
|  |  | economical vulnerability | the reciprocal of regional GDP per capita |
|  |  |  | unemployment rate |
|  | adaptability | employee's assurance | (-)number of employees joined medical assurance |
|  |  |  | (-)number of employees joined unemployment insurance |
|  |  | protection | (-)investment of infrastructure |
|  |  |  | (-)number of medical staff per thousand people |
|  |  |  | (-)number of hospital beds per thousand people |
| safety control | supervision | regulatory capacity | (-)coverage rate of supervision |
|  |  |  | (-)economic punishment |
|  |  |  | (-)punishment rate of supervision |
|  |  | personnel allocation | (-)crew size of safety supervision system |
|  |  |  | (-)number of people attending the inspection |
|  |  |  | (-)*capacity of the safety supervision crew |
|  | emergency management & publicity | emergency capacity | (-)number of fire brigade |
|  |  |  | (-)emergency resources reserves |
|  |  | safety propaganda | (-)number of news manuscripts about industrial safety |
|  |  |  | (-)*the level of public safety awareness |

* symbolizes the qualitative indexes

(-) symbolizes the negative indexes

**Part 2 Rating of index importance**

Please rate the index importance using your rich experience in Beijing industrial safety, and the importance grades are showed in table 2.

- Notice: index importance means the capability of influencing the corresponding upper level index. The more the index can influence the corresponding upper level index, the larger the index importance grade is.

Table 2. Index Importance Grades

| **Definition of different importance levels** | **Importance Grades** |
| --- | --- |
| Not important | 1 |
| Slightly important | 3 |
| Quite important | 5 |
| Obviously important | 7 |
| Absolutely important | 9 |
| Between them | 2, 4, 6, 8 |

*note: Please refer to Table 1 when you fill in the blanks to consider the hierarchy of index system.

Please fill the importance grades in all the coloured blanks with making a comparison of the indexes of the same branch (with the same colour) and the same level.

| **level 1** | **Importance Grade** | **level 2** | **Importance Grade** | **level 3** | **Importance**  **Grade** | **level 4** | **Importance**  **Grade** |
| --- | --- | --- | --- | --- | --- | --- | --- |
| disaster-inducing factors |  | accidents |  | severity |  | death toll of industrial safety issues |  |
|  |  |  |  |  |  | frequency of industrial safety issues |  |
|  |  |  |  | accountability |  | number of people investigated and affixed liability |  |
|  |  |  |  |  |  | the fines of industrial safety accidents |  |
|  |  | hidden dangers |  | number of major hazard sources | | |  |
|  |  |  |  | number of hidden dangers discovered | | |  |
|  |  |  |  | number of units with harm of occupational disease | | |  |
|  |  |  |  | number of people contacted with occupational disease | | |  |
| vulnerability of hazard-affected carriers |  | vulnerability |  | population vulnerability |  | the resident population density |  |
|  |  |  |  |  |  | proportion of aged population |  |
|  |  |  |  |  |  | proportion of children |  |
|  |  |  |  | infrastructural vulnerability |  | number of gas station per km^2^ | ------- |
|  |  |  |  | economical vulnerability |  | the reciprocal of regional GDP per capita |  |
|  |  |  |  |  |  | unemployment rate |  |
|  |  | adaptability |  | employee's assurance |  | (-)number of employees joined medical assurance |  |
|  |  |  |  |  |  | (-)number of employees joined unemployment insurance |  |
|  |  |  |  | protection |  | (-)investment of infrastructure |  |
|  |  |  |  |  |  | (-)number of medical staff per thousand people |  |
|  |  |  |  |  |  | (-)number of hospital beds per thousand people |  |
| safety control |  | supervision |  | regulatory capacity |  | (-)coverage rate of supervision |  |
|  |  |  |  |  |  | (-)economic punishment |  |
|  |  |  |  |  |  | (-)punishment rate of supervision |  |
|  |  |  |  | personnel allocation |  | (-)crew size of safety supervision system |  |
|  |  |  |  |  |  | (-)number of people attending the inspection |  |
|  |  |  |  |  |  | (-)*capacity of the safety supervision crew |  |
|  |  | emergency management & publicity |  | emergency capacity |  | (-)number of fire brigade |  |
|  |  |  |  |  |  | (-)emergency resources reserves |  |
|  |  |  |  | safety propaganda |  | (-)number of news manuscripts about industrial safety |  |
|  |  |  |  |  |  | (-)*the level of public safety awareness |  |

**Part 3 Grading of qualitative indexes**

Please give your opinion on grading the following two qualitative indexes with choosing the grade from 1 to 5 (1 means very low, 5 means very High, 3 means average).

| **district** | ***capacity of the safety supervision crew** | ***the level of public safety awareness** |
| --- | --- | --- |
| Dongcheng | □1 □2 □3 □4 □5 | □1 □2 □3 □4 □5 |
| Xicheng | □1 □2 □3 □4 □5 | □1 □2 □3 □4 □5 |
| Chaoyang | □1 □2 □3 □4 □5 | □1 □2 □3 □4 □5 |
| Haidian | □1 □2 □3 □4 □5 | □1 □2 □3 □4 □5 |
| Fengtai | □1 □2 □3 □4 □5 | □1 □2 □3 □4 □5 |
| Shijingshan | □1 □2 □3 □4 □5 | □1 □2 □3 □4 □5 |
| Fangshan | □1 □2 □3 □4 □5 | □1 □2 □3 □4 □5 |
| Tongzhou | □1 □2 □3 □4 □5 | □1 □2 □3 □4 □5 |
| Shunyi | □1 □2 □3 □4 □5 | □1 □2 □3 □4 □5 |
| Changping | □1 □2 □3 □4 □5 | □1 □2 □3 □4 □5 |
| Daxing | □1 □2 □3 □4 □5 | □1 □2 □3 □4 □5 |
| Mentougou | □1 □2 □3 □4 □5 | □1 □2 □3 □4 □5 |
| Huairou | □1 □2 □3 □4 □5 | □1 □2 □3 □4 □5 |
| Pinggu | □1 □2 □3 □4 □5 | □1 □2 □3 □4 □5 |
| Miyun | □1 □2 □3 □4 □5 | □1 □2 □3 □4 □5 |
| Yanqing | □1 □2 □3 □4 □5 | □1 □2 □3 □4 □5 |
