# Supplementary material for: Use of evidential reasoning and AHP to assess regional industrial safety
Source: PLoS One. 2018 May 24;13(5):e0197125. doi: 10.1371/journal.pone.0197125 (PMC5993124; doi:10.1371/journal.pone.0197125)
Supplement: S2 Questionnaire — (DOCX) [file pone.0197125.s005.docx]

**Supporting information**

S2 Questionnaire. Data

**Part 1 Importance Grade of Each Index**

| **Num. of Experts** | **Importance Grade** | | | | | | | | | | **Average** |
| --- | --- | --- | --- | --- | --- | --- | --- | --- | --- | --- | --- |
|  | I | II | III | IV | V | VI | VII | VIII | IX | X |  |
| **Level 1** | | | | | | | | | | | |
| disaster-inducing factors | 8 | 9 | 7 | 7 | 9 | 9 | 7 | 9 | 8 | 7 | 8.0000 |
| vulnerability of hazard-affected carriers | 9 | 7 | 3 | 8 | 7 | 9 | 5 | 9 | 8 | 7 | 7.0000 |
| safety control | 7 | 7 | 5 | 6 | 9 | 7 | 6 | 8 | 8 | 7 | 7.0000 |
| **Level 2** | | | | | | | | | | | |
| accidents | 7 | 9 | 5 | 8 | 9 | 7 | 8 | 9 | 9 | 8 | 8.0000 |
| hidden dangers | 8 | 9 | 9 | 9 | 5 | 9 | 9 | 9 | 8 | 7 | 8.2222 |
| vulnerability | 6 | 7 | 5 | 8 | 7 | 9 | 5 | 9 | 6 | 8 | 7.1111 |
| adaptability | 7 | 5 | 3 | 8 | 5 | 7 | 7 | 8 | 7 | 6 | 6.2222 |
| supervision | 7 | 9 | 7 | 5 | 9 | 7 | 7 | 8 | 7 | 6 | 7.2222 |
| emergency management & publicity | 8 | 7 | 5 | 9 | 7 | 7 | 5 | 9 | 6 | 6 | 6.7778 |
| **Level 3** | | | | | | | | | | | |
| severity | 8 | 7 | 3 | 9 | 9 | 9 | 8 | 9 | 9 | 8 | 7.8889 |
| accountability | 9 | 5 | 5 | 1 | 5 | 7 | 6 | 7 | 9 | 8 | 5.8889 |
| population vulnerability | 7 | 7 | 3 | 6 | 9 | 7 | 8 | 8 | 6 | 5 | 6.5556 |
| infrastracture vulnerability | 9 | 7 | 5 | 9 | 5 | 7 | 6 | 9 | 6 | 6 | 6.6667 |
| economical vulnerability | 7 | 7 | 3 | 8 | 7 | 7 | 7 | 7 | 6 | 4 | 6.2222 |
| employee's assurance | 8 | 5 | 3 | 6 | 5 | 7 | 5 | 7 | 6 | 4 | 5.3333 |
| protection | 9 | 5 | 5 | 9 | 7 | 7 | 7 | 8 | 6 | 5 | 6.5556 |
| regulatory capacity | 9 | 7 | 7 | 5 | 7 | 7 | 7 | 8 | 6 | 5 | 6.5556 |
| personnel allocation | 8 | 9 | 5 | 5 | 9 | 7 | 7 | 9 | 6 | 5 | 6.8889 |
| emergency capacity | 8 | 7 | 5 | 9 | 7 | 7 | 7 | 9 | 7 | 6 | 7.1111 |
| safety propaganda | 9 | 7 | 5 | 9 | 5 | 9 | 5 | 9 | 6 | 6 | 6.7778 |
| **Level 4** | | | | | | | | | | | |
| death toll of industrial safety issues | 9 | 7 | 1 | 9 | 9 | 7 | 9 | 9 | 9 | 7 | 7.4444 |
| frequency of industrial safety issues | 9 | 5 | 5 | 1 | 9 | 9 | 7 | 8 | 7 | 6 | 6.3333 |
| number of people investigated and affixed liability | 9 | 5 | 5 | 1 | 7 | 9 | 7 | 7 | 5 | 4 | 5.5556 |
| the fines of industrial safety accidents | 8 | 3 | 3 | 7 | 7 | 5 | 7 | 5 | 5 | 4 | 5.1111 |
| number of major hazard sources | 8 | 9 | 5 | 1 | 5 | 9 | 8 | 8 | 7 | 5 | 6.3333 |
| number of hidden dangers discovered | 8 | 7 | 3 | 2 | 5 | 7 | 9 | 5 | 7 | 4 | 5.4444 |
| number of units with harm of occupational disease | 7 | 7 | 3 | 8 | 7 | 9 | 6 | 7 | 7 | 5 | 6.5556 |
| number of people contacted with occupational disease | 8 | 5 | 5 | 3 | 7 | 7 | 7 | 3 | 8 | 4 | 5.4444 |
| the resident population density | 6 | 5 | 5 | 8 | 7 | 9 | 7 | 9 | 7 | 5 | 6.8889 |
| proportion of aged population | 6 | 3 | 1 | 5 | 5 | 7 | 8 | 7 | 6 | 5 | 5.2222 |
| proportion of children | 5 | 5 | 1 | 5 | 5 | 7 | 9 | 8 | 6 | 5 | 5.6667 |
| the reciprocal of regional GDP per capita | 8 | 5 | 3 | 9 | 5 | 5 | 3 | 7 | 5 | 4 | 5.1111 |
| unemployment rate | 6 | 7 | 5 | 8 | 7 | 7 | 5 | 7 | 6 | 3 | 6.1111 |
| number of employees joined medical assurance | 7 | 9 | 5 | 7 | 5 | 7 | 8 | 8 | 5 | 3 | 6.3333 |
| number of employees joined unemployment insurance | 7 | 9 | 3 | 5 | 5 | 7 | 6 | 7 | 5 | 3 | 5.5556 |
| investment of infrastructure | 8 | 7 | 5 | 9 | 5 | 7 | 7 | 7 | 6 | 5 | 6.4444 |
| number of medical staff per thousand people | 7 | 7 | 3 | 8 | 7 | 7 | 6 | 7 | 6 | 4 | 6.1111 |
| number of hospital beds per thousand people | 8 | 5 | 3 | 8 | 7 | 7 | 6 | 7 | 6 | 4 | 5.8889 |
| coverage rate of supervision | 8 | 5 | 7 | 2 | 9 | 9 | 8 | 8 | 6 | 4 | 6.4444 |
| economic punishment | 9 | 5 | 3 | 9 | 7 | 7 | 5 | 8 | 5 | 4 | 5.8889 |
| punishment rate of supervision | 9 | 5 | 3 | 9 | 7 | 7 | 6 | 7 | 5 | 3 | 5.7778 |
| crew size of safety supervision system | 7 | 5 | 7 | 4 | 9 | 7 | 6 | 9 | 7 | 5 | 6.5556 |
| number of people attending the inspection | 6 | 5 | 3 | 3 | 7 | 5 | 7 | 9 | 6 | 4 | 5.4444 |
| capacity of the safety supervision crew | 6 | 5 | 5 | 8 | 7 | 7 | 8 | 9 | 7 | 6 | 6.8889 |
| number of fire brigade | 7 | 5 | 5 | 9 | 7 | 7 | 8 | 9 | 6 | 5 | 6.7778 |
| emergency resources reserves | 6 | 5 | 3 | 8 | 5 | 5 | 5 | 9 | 6 | 5 | 5.6667 |
| number of news manuscripts about industrial safety | 7 | 5 | 3 | 9 | 7 | 9 | 5 | 9 | 5 | 5 | 6.3333 |
| the level of public safety awareness | 8 | 9 | 3 | 9 | 5 | 9 | 7 | 9 | 6 | 6 | 7.0000 |

**Part 2 Qualitative Index Data**

**1.** **capacity of the safety supervision crew**

| Num. of Experts | A | B | C | D | E | F | G | H | I | J | K | L | M | N | O | P |
| --- | --- | --- | --- | --- | --- | --- | --- | --- | --- | --- | --- | --- | --- | --- | --- | --- |
| I | 5 | 4 | 5 | 4 | 5 | 3 | 4 | 5 | 5 | 3 | 4 | 4 | 4 | 4 | 4 | 4 |
| II | 3 | 3 | 3 | 3 | 3 | 3 | 3 | 3 | 3 | 3 | 3 | 3 | 3 | 3 | 3 | 3 |
| III | 4 | 4 | 4 | 4 | 4 | 4 | 4 | 4 | 4 | 4 | 4 | 4 | 4 | 4 | 4 | 4 |
| IV | 4 | 4 | 3 | 2 | 3 | 2 | 3 | 2 | 3 | 2 | 2 | 2 | 2 | 2 | 2 | 2 |
| V | 5 | 4 | 4 | 4 | 4 | 4 | 3 | 4 | 4 | 3 | 3 | 3 | 3 | 3 | 3 | 3 |
| VI | 5 | 5 | 5 | 5 | 4 | 4 | 4 | 5 | 4 | 5 | 5 | 4 | 5 | 4 | 4 | 4 |
| VII | 4 | 4 | 4 | 4 | 4 | 4 | 4 | 4 | 4 | 4 | 4 | 4 | 4 | 4 | 4 | 4 |
| VIII | 5 | 5 | 5 | 5 | 5 | 5 | 5 | 5 | 5 | 5 | 5 | 5 | 5 | 5 | 5 | 5 |
| IX | 5 | 5 | 5 | 5 | 4 | 4 | 4 | 5 | 4 | 4 | 4 | 4 | 4 | 4 | 4 | 4 |
| X | 4 | 4 | 4 | 4 | 4 | 3 | 4 | 4 | 4 | 4 | 3 | 3 | 4 | 3 | 4 | 3 |
| FBS | (0,0,0.1,0.4,0.5) | (0,0,0.1,0.6,0.3) | (0,0,0.2,0.4,0.4) | (0,0.1,0.1,0.5,0.3) | (0,0,0.2,0.6,0.2) | (0,0.1,0.3,0.5,0.1) | (0,0,0.3,0.6,0.1) | (0,0.1,0.1,0.4,0.4) | (0,0.2,0.6,0.2） | (0,0.1,0.3,0.4,0.2) | (0,0.1,0.3,0.4,0.2) | (0,0.1,0.3,0.5,0.1) | (0,0.1,0.2,0.5,0.2) | (0,0.1,0.3,0.5,0.1) | (0,0.1,0.2,0.6,0.1) | (0,0.1,0.3,0.5,0.1) |

**2. the level of public safety awareness**

| Num. of Experts | A | B | C | D | E | F | G | H | I | J | K | L | M | N | O | P |
| --- | --- | --- | --- | --- | --- | --- | --- | --- | --- | --- | --- | --- | --- | --- | --- | --- |
| I | 4 | 4 | 3 | 5 | 3 | 4 | 4 | 4 | 3 | 4 | 2 | 3 | 3 | 3 | 3 | 4 |
| II | 4 | 4 | 4 | 4 | 3 | 3 | 3 | 3 | 3 | 3 | 3 | 3 | 3 | 3 | 3 | 3 |
| III | 4 | 3 | 4 | 4 | 4 | 4 | 4 | 4 | 4 | 4 | 4 | 4 | 4 | 4 | 4 | 4 |
| IV | 2 | 4 | 4 | 3 | 1 | 2 | 2 | 2 | 2 | 1 | 1 | 1 | 1 | 1 | 1 | 1 |
| V | 5 | 5 | 5 | 4 | 4 | 4 | 4 | 4 | 3 | 3 | 3 | 3 | 3 | 3 | 3 | 3 |
| VI | 5 | 5 | 4 | 4 | 3 | 4 | 3 | 4 | 4 | 3 | 3 | 3 | 3 | 3 | 3 | 3 |
| VII | 4 | 4 | 4 | 4 | 4 | 4 | 4 | 4 | 4 | 4 | 4 | 4 | 4 | 4 | 4 | 4 |
| VIII | 5 | 5 | 4 | 5 | 4 | 5 | 5 | 5 | 5 | 4 | 4 | 4 | 4 | 4 | 4 | 4 |
| IX | 5 | 5 | 5 | 5 | 4 | 4 | 4 | 4 | 4 | 4 | 4 | 4 | 4 | 4 | 4 | 4 |
| X | 3 | 3 | 3 | 3 | 3 | 3 | 3 | 3 | 3 | 3 | 3 | 3 | 3 | 3 | 3 | 3 |
| FBS | (0,0.1,0.1,0.4,0.4) | (0,0,0.2,0.4,0.4) | (0,0,0.2,0.6,0.2) | (0,0,0.2,0.5,0.3) | (0.1,0,0.4,0.5,0) | (0,0.1,0.2,0.6,0.1) | (0,0.1,0.3,0.5,0.1) | (0,0.1,0.2,0.6,0.1) | (0,0.1,0.4,0,4,0.1) | (0.1,0,0.4,0.5,0) | (0.1,0.1,0.4,0.4,0) | (0.1,0,0.5,0.4,0) | (0.1,0,0.5,0.4,0) | (0.1,0,0.5,0.4,0) | (0.1,0,0.5,0.4,0) | (0.1,0,0.4,0.5,0) |
